# Chemical Diversity in Species Belonging to Soft Coral Genus *Sacrophyton* and Its Impact on Biological Activity: A Review

**DOI:** 10.3390/md18010041

**Published:** 2020-01-06

**Authors:** Yasmin A. Elkhawas, Ahmed M. Elissawy, Mohamed S. Elnaggar, Nada M. Mostafa, Eman Al-Sayed, Mokhtar M. Bishr, Abdel Nasser B. Singab, Osama M. Salama

**Affiliations:** 1 Department of Pharmacognosy and Medicinal plants, Faculty of Pharmaceutical Sciences and Pharmaceutical Industries, Future University in Egypt, Cairo 11835, Egypt; osalama@fue.edu.eg; 2 Department of Pharmacognosy, Faculty of Pharmacy, Ain-Shams University, Cairo 11566, Egypt; aelissawy@pharma.asu.edu.eg (A.M.E.); mohamed.s.elnaggar@pharma.asu.edu.eg (M.S.E.); nadamostafa@pharma.asu.edu.eg (N.M.M.); em_alsayed@pharma.asu.edu.eg (E.A.-S.); dean@pharma.asu.edu.eg (A.N.B.S.); 3 Center of Drug Discovery Research and Development, Ain-Shams University, Cairo 11566, Egypt; 4 Plant General Manager and Technical Director, Mepaco Co., Sharkeiya 11361, Egypt; mbishr_2000@yahoo.com

**Keywords:** *Sacrphyton*, soft coral, terpenoids, antimicrobial, antitumor, antidiabetic, anti-inflammatory

## Abstract

One of the most widely distributed soft coral species, found especially in shallow waters of the Indo-Pacific region, Red Sea, Mediterranean Sea, and also the Arctic, is genus *Sacrophyton*. The total number of species belonging to it was estimated to be 40. *Sarcophyton* species are considered to be a reservoir of bioactive natural metabolites. Secondary metabolites isolated from members belonging to this genus show great chemical diversity. They are rich in terpenoids, in particular, cembranoids diterpenes, tetratepenoids, triterpenoids, and ceramide, in addition to steroids, sesquiterpenes, and fatty acids. They showed a broad range of potent biological activities, such as antitumor, neuroprotective, antimicrobial, antiviral, antidiabetic, antifouling, and anti-inflammatory activity. This review presents all isolated secondary metabolites from species of genera *Sacrophyton*, as well as their reported biological activities covering a period of about two decades (1998–2019). It deals with 481 metabolites, including 323 diterpenes, 39 biscembranoids, 11 sesquiterpenes, 53 polyoxygenated sterols, and 55 miscellaneous and their pharmacological activities.

## 1. Introduction

**Figure 1:**
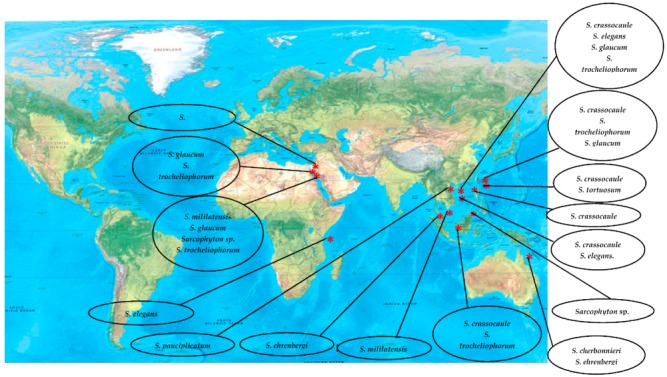
Worldwide distribution of chemically studied *Sarcophyton* soft coral.

**Figure 2:**
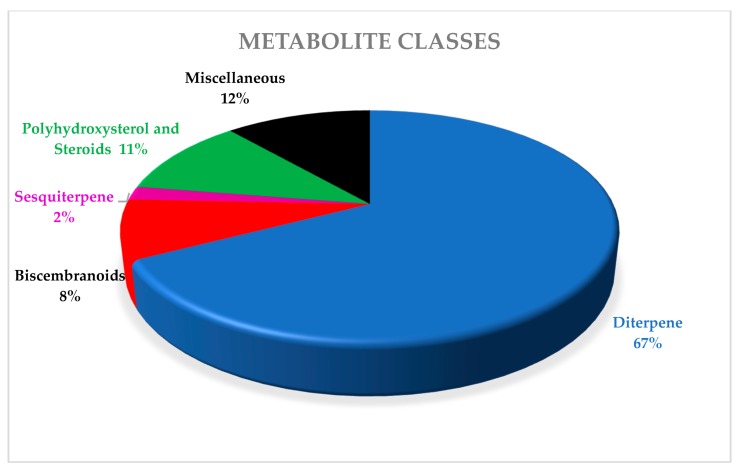
Pie chart showing the percentage of each class of metabolites identified in Sarcophyton sp.

**Figure 3:**
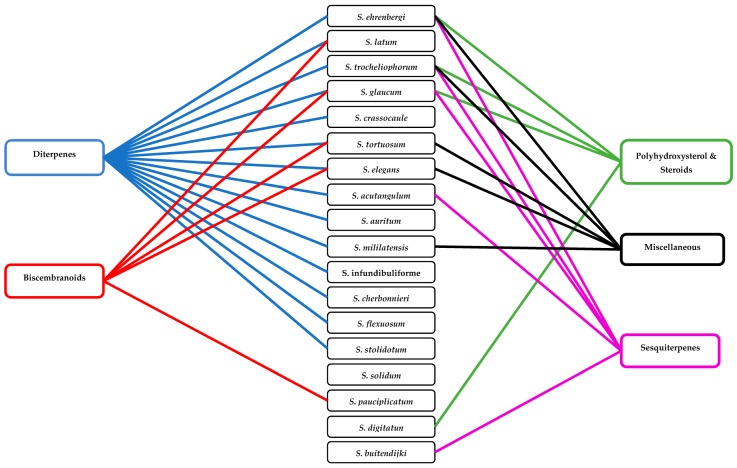
A diagram of isolated classes from each *Sarcophyton* sp.

Classification of alcyonacean corals, subclass Octocorallia implies the existence of polyps with eight tentacles, which differentiates them from hexacorallian Scleractinia corals. Alcyonaceans are sessile large invertebrate with distinct stalk and a smooth, mushroom-shaped top known as capitulum, and their tissue comprises sclerites, which give support to the colony [1,2]. Traditionally, identification and classification of most soft coral have been carried out by sclerite classification. *Sarcophyton* covers 35 species, and another six species of *Sarcophyton* were described [3,4,5,6,7,8]. Later, [9] reported that, within *Sarcophyton* samples, *Sarcophyton glaucum* contains six different genetic clades, signifying that this morphologically heterogeneous species was mysterious [10]. Studies revealed that *Sarcophyton* were mostly seen in shallow water of the Indo-Pacific region [11,12], Red Sea [13], Mediterranean Sea [14], and also the Arctic area [10,15]. However, to our knowledge, nothing was reported from North and South of America (Figure 1). *Sarcophyton* sp. synonyms include Toadstool Mushroom Leather, Toadstool Leather Coral, Umbrella Coral, Toadstool Mushroom Coral, Mushroom Leather Coral, *Sarcophyton* Coral, and Mushroom Coral. *Sarcophyton* sp. were considered a reservoir of bioactive natural metabolites such as diterpenes, steroids, sesquiterpenes, and fatty acids [16,17]. These metabolites, mainly macrocyclic cembranes and their byproducts, represented an important natural bioactive product, with significant biological activities, including anticancer [18,19], antimicrobial [20], anti-inflammatory [21], anti-osteoporotic, antimetastatic, antiangiogenic, and neuroprotective [22]. One metabolite, sarcophytol A **15**, isolated from *Sarcophyton* obtained from Ishigaki Island, Okinawa, Southern Japan, was studied and highlighted because of its important anticancer activity [23]. Some recent articles had partially covered the chemistry and pharmacology of secondary metabolites from *Sarcophyton* sp. [24,25,26]. This review concentrates on marine bioactive metabolites isolated from *Sarcophyton* species, their biological properties, and studies of the biosynthesis of marine metabolites. In this review, we reported all metabolites isolated from *Sarcophyton* species and their reported biological activities stated in the literature over the years from 1998 to 2019. Different online databases were utilized through this review, including Scifinder, Marinlit, and Web of Science. The present review aims to present the progress made in the last two decades regarding the potential application of biomolecules (481 compounds) isolated from *Sacrophyton* soft corals, to complete the previously published papers (Figure 2 and Figure 3) on the interesting subject of *Sacrophyton*. It deals with the chemistry, as well as the biological activity of secondary metabolites, including terpenoids, in particular diterpenes, sesquiterpenes, biscembranoids, and polyhydroxysterols, in addition to a number of miscellaneous compounds. The percentage of different chemical classes is represented in (Figure 2), and Figure 3 shows a diagram of isolated classes from each *Sarcophyton* sp.

## 2. Classes of Secondary Metabolites

### 2.1. Diterpenes

*Sarcophyton ehrenbergi* dichloromethane extract yielded sarcophytol T **1**, (1*E*,3*E*,7*E*,11R*12R*)-15-(acetoxymethyl)cembra-11,12-epoxy-1,3,7-triene **2**, and (11S*,12S*)-15-(acetoxymethyl) cembra-3,4:11,12-diepoxy-1,7-diene **3**, together with known isoneocembrene A **4**, an isomer to neocembrane A **5**, and (2S*,11R*,12R*)-isosarcophytoxide **6**. Compound **2** was found to possess several structural similarities with the former two isolates in conjugated diene system (C-1 and C-4) and Δ^7,8^ double bond and 11,12-epoxy functional group [27]. 

Another three cembrenolide diterpenes identified as crassolide **7**, sarcocrassolide A **8**, and 13-acetoxysarcocrassolide **9**, alongside known cembrenolide denticulatolide **10**, were reported from *S. crassocaule* [28].

From *S. trocheliophorum*, the isolation of 7,8-epoxy-1(*E*),3(*E*),11(*E*)cembratrien-15-ol **11**, 7,8-epoxy1(*E*),3(*E*),11(*E*)-cembratriene **12**, and sarcophin **13** was reported, and the absolute configuration of sarcophin **13** was investigated through modified Mosher’s assay [29].

Using chromatographic techniques, cembrane alcohol identified as acutanol **14** beside sarcophytol A **15** and sarcophytol A acetate **16** were isolated from *S. acutangulum* extract. The absolute configuration of sarcophytol A **15** was assessed with the use of many chiral anisotropic reagents, as 1-naphthylmethoxyacetic acid [30].

Four cembranes, (1*S*,2*E*,4*R*,6*E*,8*S*,11*S*,12*S*)-11,12-Epoxy-2,6-cembrane-4,8-diol **17**, (1*S*,2*E*,4*R*,6*E*,8*R*,11*S*,12*S*)-11,12-Epoxy-2,6-cembrane-4,8-diol **18**, (1*S*,2*E*,4*R*,7*S*)-11,12-Epoxy-2,8(19)-cembradiene-4,7-diol **19**, and (1*S*,2*E*,4*R*,7*R*)-11,12-Epoxy-2,8(19)-cembradiene-4,7-diol **20**, were isolated from *Sarcophyton* sp. It is worth noticing that these metabolites were not previously found in nature. Their absolute configurations were validated with X-ray analysis [31].

The hydroperoxide cembrane diterpenoid, sarcophycrassolide A **21**, together with sacophyocrassolide B **22** and compound **8**, was reported from *S. crassocaule*. Identification of compound **21** was resolved by using X-ray diffraction and spectral analysis [32].

Three furano-cembranoids and two seco-cembranoid acetates, which were identified as 13-dehydroxysarcoglaucol **23**, 13-dehydroxysarcoglaucol-16-one **24** and sarcoglaucol-16-one **25**, (3*E*)-7-hydroxy-4,8,15,15-tetramethyl-1-[(*E*)-12-methyl-10-oxo-12-pentenyl]-3,8-decadienyl acetate **26**, (3*E*)-7-hydroxy-4,8,15,15-tetramethyl-1-[(*Z*)-12-methyl-10-oxo-12-pentenyl]-3,8-decadienyl acetate **27** beside sarcoglaucol **28**, and decaryiol **29**, were isolated form *S. cherbonnieri*. Spectral data showed that compound **25** was a 16-keto derivative of compound **28** and the 13-hydroxy derivative of compound **24** [33]. The absolute configuration of compound **25** was investigated similarly to compound **13** by using the modified Mosher’s method [34]. Another two bicyclic cembranolides metabolites, with infrequent structures in marine literature, having a 12*Z* double bond, identified as (4*Z*,8*S*,9*S*,12*Z*,14*E*)-9-Hydroxy-1-isopropyl-8,12-dimethyl-oxabicyclo [9.3.2]-hexadeca-4,12,14-trien-18-one **30**, and (4*Z*,12*Z*,14*E*)-sarcophytolide **31**, in addition to sarcophytolide **32**, (4*Z*,8*S*,9*R*,12*E*,14*E*)-9-Hydroxy-1-isopropyl-8,12-dimethyloxabicyclo[9.3.2]-hexadeca-4,12,14-trien-18-one **33** and (4*Z*,8*S*,9*R*,12*E*,14*E*)-1-Isopropyl-8,12-dimethyl-18-oxo-oxabicyclo[9.3.2]-hexadeca-4,12,14-trien-2-yl acetate **34**, were reported from *Sarcophyton* new sp. Additionally, the authors presented biosynthetic pathways for all isolated compounds which resulted from the common acyclic precursor (all-*E*)-geranylgeranyl pyrophosphate (GGPP), by converting geranylgeranyl-PP [GGPP] into geranylneryl-PP [GNPP], using diterpene synthase, followed by cyclization to cembranoid ring with a 12*Z* double bond [35]. Three diterpenes, sarcophytolol **35**, sarcophytolide B **36**, and sarcophytolide C **37**, were reported from *S. glaucum* [34].

Sarcophytonolides A–D **38**–**41**, four cembranolides were isolated from *S. tortuosum*. Sarcophytonolide B **39** was found to be the 12-(methoxycarbonyl) derivative of compound **38**, in which it exhibited αβ-unsaturated methyl ester instead of the methyl group. Sarcophytonolide D **41** was similar in structure to compound **40**, while, compound **41** possessed an extra trisubstituted C=C and acetoxy group [36]. Four more sarcophytonolides E-H **42**–**45** from *S. latum* were isolated. All isolated compounds were related in structure to compound **40**, with an α, β-unsaturated butanolide group. Sarcophytonolide G **44** was found to be the epimer of Sarcophytonolide F **43** at C-6, while sarcophytonolide H **45** was 14-acetoxy methoxycarbonyl derivative of compound **43**. The absolute configuration was investigated by using the modified Mosher’s assay as they all possessed secondary alcohol at C-6. It is worth noting that the structural configuration supporting all cembrane diterpenes stated, in the order alcyonacea, with the identified absolute configuration at C-1, belonged to α-series [37]. Moreover, sarcophytonolides I–L **46**–**49** were isolated from *S. latum*. All compounds were related in structure to the previously isolated compounds **38**–**45**; all possessed α, β-unsaturated butenolactone group. The absolute configuration of compounds **38**–**45** still need further determination. Considering the fact that these compounds were structurally related to previously isolated sarcophytonolide, the structure of sarcophytonolide I **46** differs from sarcophytonolide D **41**, in the olefinic C_7_=C_8_ bond and absence of C=O group at C6 [38]. Another five cembranolide, sarcophytonolides N–R **50**–**54**, ketoemblide **55**, and (*E*,*E*,*E*)-1-isopropenyl-4,8,12-trimethylcyclotetradeca-3,7,11-tiene **56** were isolated from *S. trocheliophorum* Marenzeller. A detailed spectroscopic analysis was done, in which sarcophytonolides N–R **50**–**54** were found to be either mono- or bicyclic cembranoids possessing oxidized methyl groups and three/four double bonds [39]. The absolute configuration of another six metabolites isolated from *S. trocheliophorum*, sarcophytonolides S–U **57**–**59** and sartrolides H–J; α,β-unsaturated ε-lactone **60**–**62**, along with seven known analogues, were carried out through different techniques [40]. Chemical determination of *S. trocheliophorum* yielded seven cembranolides, sartrolides A–G **63**–**69** and bissartrolide dimer **70**; a third member of this scarce class of cembrane dimers [41]. Yalongenes A and B **71** and **72** another two cembranoids, isolated from *S. trocheliophorum* [42], and another two cembranoids, trochelioids A and B **73** and **74,** and 16-oxosarcophytonin E **75** were isolated [43].

Five diterpenes cembrane type, sarcrassins A–E **76**–**80**, beside emblide **81** isolated from *S. crassocaule* were identified based on 1D and 2D NMR. Sarcrassins B and C **77** and **78**, cyclic diterpenes, derivatives of sarcrassin A **76** in which the double bond in sarcrassin A **76** was replaced by an epoxy ring in sarcrassin B **77**. However, in sarcrassin C **78** the epoxy ring in sarcrassin A **76** was replaced by a hydroxyl and methoxy group. As for sarcrassin D **79**, its bicyclic diterpene structure was confirmed through spectral data [44], and its absolute configuration, as well as that of emblide **81,** was determined by X-ray analysis [41,45].

Investigation of ethyl acetate extract of *S. crassocaule* yielded six polyoxygenated cembrane-diterpenoids with a trans-fused α-methylene-γ-lactone, identified as crassocolides A–F **82**–**87** alongside lobophytolide **88**. Absolute configuration for crassocolide A **82** was resolved by using modified Mosher’s method [46]. Another seven polyoxygenated cembranoids with α-methylene-γ-lactone group identified as crassocolides G–M **89**–**95**, were reported. The structures of all compounds were determined through a full spectral data analysis, and the absolute configuration of crassocolide G **89** was investigated by modified reaction of Mosher’s assay [47]. Other crassocolides N–P **96**–**98** were isolated *from S. crassocaule* [48]. The CHCl_3_/MeOH extract of *S. flexuosum* yielded three cembranes, identified through spectral data as flexusines A, B, and epimukulol **99**–**101** [49].

From ethyl acetate extract of *S. stolidotum*, seven cembranes, sarcostolides A–G **102**–**108**, alongside isosarcophin **109**, were reported, and their structures were elucidated through spectral data. The authors also proposed a reasonable biogenetic pathway for all isolates, in which cyclization of GPP with lactonization and oxidation may lead to the production of sarcostolide C **104**. Sarcostolides A and B **102** and **103** and D–G **105**–**108** were converted from sacostolide C **104** through migration and isomerization of double bonds [50].

*Sarcophyton mililatensis* methanol extract yielded cembranoid diterpenes identified as (−)-7β-hydroxy-8α-methoxy-deepoxy-sarcophytoxide **110**, (−)-7β,8β-dihydroxy-deepoxy-sarcophytoxide **111**, (−)-17-hydroxysarcophytonin A **112**, sarcophytol V **113**, and sarcophytoxide **114** [51].

Two cembrane diterpenes known as 17-hydroxysarcophytoxide **115** and 7β-acetoxy-8α-hydroxydeepoxysarcophine **116**, along with 7β,8α, dihydroxydeepoxysarcophine **117**, sarcophytonin A **118**, and (−)-β-elemene **119** reported from *Sarcophyton* sp., were isolated from *S. glaucum* [52]. Investigation of *S. glaucum* extract led to the isolation of two cembranoids, (7*R*,8*S*)-dihydroxydeepoxy-ent-sarcophine **120** and secosarcophinolide **121**, in addition to, ent-sarcophin **122**. Structural elucidation of the isolates was established by their spectral data and chemical correlation, as (7*R*,8*S*)-dihydroxydeepoxy-ent-sarcophine **120** was found to be the enantiomer of (7*S*,8*R*)-dihydroxydeepoxysarcophine **123** and compound **121** has a unique butyl ester group at C-16 [53].

Seven cembranoids were isolated from *Sarcophyton* sp., 5-epi-sinuleptolide **124**, lobohedleolide **125**, (7*Z*)-lobohedleolide **126**, and two uncommon cembranoids, sarcofuranocembrenolide A **127**; with a unique carbon skeleton of 8,19-bisnorfuranocembrenolide, and sarcofuranocembrenolide B **128**; a furanocembrenolide [54]. Sarcophytonins F and G **129** and **130**, another two dihydrofuranocembranoids, were reported from *Sarcophyton* sp. [55]. Nineteen compounds from *Sarcophyton* sp., of which five cembrane diterpenoids were isolated and identified as 7-acetyl-8-epi- sinumaximol G **131**, 8-epi- sinumaximol G **132**, 12-acetyl-7,12-epi- sinumaximol G **133**, 12-hydroxysarcoph-10-ene **134**, and 8-hydroxy-epi-sarcophinone **135**, together with sinumaximol G **136**, were reported [56].

Five isolated cembranoids, sarcocrassocolides A–E **137**–**141**, together with sinularolide **142**, were isolated from *S. crassocaule*. Structural elucidation of the compounds was determined through spectral analysis, and the absolute configuration of sarcocrassocolide A **137** was investigated by modified Mosher’s method. It is worth mentioning that sarcocrassocolides A–D **137**–**140** possessed a tetrahydrofuran group with a seldomly found 4,7-ether bond, which was discovered previously in *Eunicea mammosa* soft coral [57,58]. Another seven cembranoids with α-methylene-γ-lactonic group and rare trans 6,7-disubstituted double bond, uncovered earlier only in soft coral *Eunicea pinta*, identified as sarcocrassocolides F–L **143**–**149**, were isolated from *S. crassocaule* [59]. Besides the abovementioned sarcocrassocolides, another three sarcocrassocolides, M–O **150**–**152**, from *S. crassocaule*, were identified. Through structural analysis, sarcocrassocolide N **151** was found to have the same relative configuration of sarcocrassocolide M **150**, while sarcocrassocolide O **152** was found to be the 13-deacetoxy derivative of sarcocrassocolide M **150** [60]. Three more cembranoids, sarcocrassocolides P–R **153**–**155**, were identified, and their structures were investigated by an extensive spectral study [61].

Investigation of n-hexane fraction for *S. ehrenbergi* led to the isolation of (+)-7,8-epoxy-7,8-dihydrocembrene C **156**, in which its optical rotation indicated that it was (+)- (7*S*,8*S*)-7,8-epoxy-7,8-dihydrocembrene C **156**, not (−)-7,8-Epoxy-7,8-dihydrocembrene C, which was reported previously from *S. crassocaule* [62].

Six cembranoids, (+)-12-carboxy-11*Z*-sarcophytoxide **157**, (+)-12-methoxycarbonyl-11*Z*-sarcophine **158**, ehrenberoxides A–C **159**–**161** and lobophynin C **162** were isolated from *S. ehrenbergi*. Compound **157** has a 2,5-dihydrofuran ring attached to a 14 membered ring at carbon-1 and carbon-2, a carboxylic acid at carbon-12 and an epoxide moiety at carbon-7 and carbon-8. Moreover, the authors mentioned that both ehrenberoxides B and C **160**–**161** raised from the exact precursor with a 7,8-epoxide through a transannular cleavage of the 7,8-epoxide by both ends of an 11,12-diol, while compound **160** has a unique oxepane ring, which was not detected previously in cembranoid [63] and from *S. infundibuliforme* diterpenoids cembrene C **163**, sarcophytol B **164**, sarcophytol E **165**, and sarcophytol H **166**, (−)-marasol **167** were reported [64].

A cembrane diterpene identified as 2*R*,7*R*,8*R*-dihydroxydeepoxysarcophine **168** was isolated from *S. glaucum* [65], and three compounds were reported from its ethyl acetate fraction, of which two were peroxide diterpenes identified as 11(*S*)-hydroperoxylsarcoph-12(20)-ene **169**, 12(*S*)-hydroperoxylsarcoph-10-ene **170**, and 8-epi-sarcophinone **171**. All structures were investigated by spectral data, and their relative configuration was assigned by X-ray diffraction [66].

Methyl sarcotroates A and B **172** and **173** two diterpenes, along with sarcophytonolide M **174**, a precursor for the former two compounds, were isolated from *S. trocheliophorum*, and their biogenetic pathways were proposed, in which isomaration, cycloaddition followed by oxidation of compound **174** led to the formation of both compounds **172** and **173**. The authors also studied the absolute configuration of methyl sarcotroate B **173** through TDDFT ECD calculations, helping in determining the absolute configurations for methyl sarcotroate A **172** and sarcophytonolide M **174** by a biogenetic relationship and ECD comparison, respectively [67].

Cembranoid diterpene, identified as (1*S*,2*E*,4*R*,6*E*,8*S*,11*R*,12*S*)-8,11-epoxy-4,12-epoxy- 2,6-cembradiene **175**, (1*S*,2*E*,4*R*,6*E*,8*R*,11*S*,12*R*)-8,12-epoxy-2,6-cembradiene-4,11-diol **176**, and (1*S*,4*R*,13*S*)-cembra-2*E*,7*E*,11*E*-trien-4,13-diol **177**, were reported from nature for the first time, from *S. glaucum* [68].

From an acetone extract of *S. ehrenbergi*, three cembranoids were isolated. Through full NMR data, the existence of α, β unsaturated ethyl ester and α, β unsaturated methyl ester of both (+)-12-ethoxycarbonyl-11*Z*-sarcophine; ehrenbergol A and B **178**–**180** were confirmed. Ehrenbergol B **179** showed a trisubstituted epoxide and two trisubstituted olefins. [69].

Fifteen cembrane-type diterpenoids were isolated from *S. elegans*, sarcophyolides B–E **181**–**184**, along with sarcophytol L **185**, 13α-hydroxysarcophytol L **186**, sarcophyolide A **187**, sarcophinone **188**, 7α-hydroxy-Δ8(19)-deepoxysarcophine **189**, 4β-hydroxy-Δ2(3)-sarcophine **190**, 1,15β-epoxy-2-epi-16-deoxysarcophine **191**, sarcophytol Q **192**, and lobocrasol **193**. A detailed structural elucidation was determined by spectral data and reported data. The absolute configurations of sarcophyolides B–E **181**–**184** were approved by single-crystal X-ray diffraction assay, using Flack’s assay [22], and the structure of lobocrasol **193** was further studied [70].

From the ethyl acetate extract of *S. ehrenbergi* two diterpenes were isolated, acetyl ehrenberoxide B **194** and ehrenbergol C **195**. Ehrenbergol C **195** shared a structure similar to lobocrasol **193**, isolated from *Lobophytum crassum* [71]. Yet, relative stereochemistry of carbon-7 and carbon-8 in ehrenbergol C **195** differed from lobocrasol **193** in hydroxy group and a conjugated enone evidenced by the IR spectrum at 3444 and 1696 cm^−1^, respectively [72].

An oxygenated cembranoid diterpene, sarcophytol W **196**, together with (2*E*,7*E*)-4,1l-dihydroxy-1,12-oxidocembra-2,7-dien **197**, were isolated before from *S. infundibuliforme* and *S. glaucum*, (+)-11,12-epoxy-11,12-dihydrocembrene-C **198**, (+)-11,12-epoxysarcophytol A **199** and sarcolactone A **200**, previously known, were reported from *Sarcophyton* sp. Structures were determined through spectral data and comparing the reported data. The absolute configuration of sarcophytol W **196** was elucidated based on the modified Mosher’s assay [73].

Two diterpenes were isolated from *S. tortuosum*, identified as tortuosenes A and B **201** and **202**. Structural elucidation of compounds **201** and **202** were investigated by spectral data. The absolute configuration of tortuosene A **201** was investigated using TDDFT ECD method. Moreover, the authors proposed a biosynthetic pathway for tortuosenes A and B **201** and **202** from the assumed cembranoidal precursor; (1*Z*, 3*Z*, 7*E*, 11*E*)-4-isopropyl-1,7,11-trimethylcyclotetradeca-1,3,7,11-tertaene, by oxidation of carbon-20 and the carbon-7/carbon-8 double bond was epoxidize, forming aldehydocembrane, a structure related to emblide **81**. The resulting aldehydocembrane additionally formed a cycle from carbon-2 to carbon-20 by acid-catalyzed affecting the carbon-1/carbon-2 double bond of the carbonyl moiety [74].

2-epi-sarcophine **203** and (1*R*,2*E*,4*S*,6*E*,8*R*,11*R*,12*R*)-2,6-cembradiene-4,8,11,12-tetrol **204**, two diterpenes were isolated from *S. auritum* [75]. An extensive chemical investigation of *Sarcophyton* sp. extract yielded four cembranoids, sarcophytons A–D **205**–**208**, along with cembranoids, 2-[(*E*,*E*,*E*)-7′,8′-epoxy-4′,8′,12′-trimethylcyclotetradeca-1′,3′,11-trienyl]propan-2-ol **209**, (1*E*,3*E*,7R*,8R*,11*E*)-1-(2-methoxy-propan-2-yl)-4,8,12-trimethyloxabicyclo[12.1.0]-pentadeca-1,3,11-triene **210**, crassumol C **211**, and laevigatol A **212**. [76]. Two unique pyrane-based cembranoids, sarcotrocheliol acetate and sarcotrocheliol **213** and **214** were isolated from *S. trocheliophorum* [77]. Investigation of *S. glaucum* organic extract resulted in the isolation of sarcophinediol **215**, previously processed semi-syntheticaly [78].

Cembranoid diterpenes, 7-keto-8α-hydroxy-deepoxysarcophine **216** similar to compound **13**, in which the carbon at carbon-3 and carbon-11 were presumed to be in E configuration established on compound **13** derivatives; this was established through spectral data. 7β-chloro-8α-hydroxy-12acetoxy-deepoxysarcophine **217** was close to 7-keto-8α-hydroxy-deepoxysarcophine **216** except for the disappearance of ketone signal at C-7 which co-exists with the presence of an up fielded signal at δ62.9 (C-7), a downfield of C-20 and the presence of carbonyl and methyl group at 170 and 22.2, respectively, were isolated from *S. ehrenbergi*. [79].

From *S. trocheliophorum*, sarsolenane diterpenes and capnosane diterpenes were obtained. Sarsolenane diterpenes are uncommon in nature, symbolized only by sarsolenone isolated from *S. solidum*. Two sarsolenane diterpenes, dihydrosarsolenone **218**, methyl dihydrosarsolenoneate **219**, and two capnosane diterpenes, sarsolilides B and C **220** and **221**, together with sarsolilide A **222** were isolated. Dihydrosarsolenone **218** resulting from sarsolenone **223** by terminal double bond Δ^15^ reduction followed by the oxidation of C-18 gave methyl dihydrosarsolenoneate **219**. Capnosane diterpenes were first isolated from *S. solidum* and *S. trocheliphorum*. The only example reported with α, β-unsaturated ε-lactone subunit was sarsolilide A **222**, from *S. solidum*, in which, the hydration of the exomethylene group provided carbon-10 epimers, sarsolilides B and C **220** and **221** [80]

Ethyl acetate extract of *S. trocheliophorum* yielded twenty-three isolates, of which nineteen were cembranoids with unique capnosane skeleton identified as trocheliophols A–S **224**–**242** and two analogues, 4-epi-sarcophytol L **243** and sarcophyolide C **182**. The structures were investigated by a full spectral data, and their absolute configurations were established through modified Mosher’s assay, CD and X-ray diffraction. Trocheliophols C **226**, E **228**, F **229**, and M **236** all possessed a structure similar to sarcophytolide C **176**, while, trocheliophol Q **240** was identified as the C-8 methoxylated model of trocheliophol F **229**. However, trocheliophol R **241** possessed a similar structure to trocheliophol F **229** but it differed in the presence of the methoxy group [81].

Chemical determination of *S. elegans* CH_2_Cl_2_/MeOH extract resulted in isolation of four cembranoids identified as sarcophelegans A–D **244**–**247**. Sarcophelegan A **244** was found to be the 11,12-epoxy derivative of sarcophelegan C **246**. Through X-ray crystallographic examination using anomalous scattering of Cu Kα radiation, sarcophelegan A **244** structure was verified. Moreover, sarcophelegan C **246** was found to be the 7-hydrogenated derivative of sarcophelegan B **245** [18].

Five polyoxygenated cembranoids were identified as polyoxygenated cembranoids, (+)-1,15-epoxy-2-methoxy-12methoxycarbonyl-11*E*-sarcophytoxide **248**, (+)-2-epi-12-methoxycarbonyl-11*E*-sarcophine **249**, 3,4-epoxyehrenberoxide A **250**, ehrenbergol D **251** and ehrenbergol E **252** in *S. ehrenbergi*. The authors proposed that (+)-1,15-epoxy-2-methoxy-12methoxycarbonyl-11*E*-sarcophytoxide **248** was the 1,15 epoxy-2-methoxylated equivalent of lobophynin C **162**. Through investigating the spectral data and X-ray crystallization of (+)-2-epi-12-methoxycarbonyl-11*E*-sarcophine **249** it was found that it differed in the alignment of the α,β-unsaturated γ-lactone ring attached to C-2 of the 14-membered ring [63]. 3,4-epoxyehrenberoxide A **250**; an analogue to ehrenberoxide A **159** where the epoxide in ehrenberoxide A **159** was substituted by a double bond at C3 and C4 [82].

Eight metabolites were isolated from *S. solidum*, three sarsolenanes, 7-deacetyl-sarsolenone **253**, sarsolenone **223**, and methyl dihydro-sarsolenoneate **219** together with, sarsolilide B **220**. All 7-deacetyl-sarsolenone **253**, sarsolenone **223**, sarsolilide B **220**, could be used as a chemotaxonomic marker for this species [83].

Three isolates; trocheliane **254**, tetracyclic biscembrane and two cembranoid diterpenes, sarcotrocheldiols A and B **255** and **256**, were isolated from *S. trocheliophorum*. Their relative configuration and structure of the isolates were investigated by spectral data [84].

From *Sarcophyton* sp., one cembrane diterpene, 16-hydroxycembra-1,3,7,11-tetraene **257**, besides, 15-hydroxycembra-1,3,7,11-tetraene **258** were reported. Structures were investigated by spectral data [85].

Three cembranoids from *S. trocheliophorum*, sarcophytrols D–F **259**–**261** highly oxidative compounds, besides, 11,12-epoxy-1(*E*),3(*E*), 7(*E*)-cembratrien-15-ol **262** and sinugibberol **263** were isolated. All structures were investigated by a full spectral data and by comparing with previous stated data [86]. Another six cembranoids, sarcophytrols G–L **264**–**269** together with crassumol A **270**, were isolated from *S. trocheliophorum* [87]. Additionally, another nine cembranoids, sarcophytrols M–U **271**–**279**, were also reported. Their structures were interpreted with extensive spectral analysis and chemical conversion and the absolute configuration for sarcophytrols M–S **271**–**277** were investigated by the modified Mosher’s assay. Sarcophytrols R and S **276** and **277** revealed a unique decaryiol skeleton with an uncommon C12/C15 cyclization [88]. Another cembranoid, trocheliolide B **280** from *S. trocheliophorum* was isolated [89]. Chemical determination of *S. trocheliophorum* organic extract, yielded pyrane-based diterpene, 9-Hydroxy-10,11-dehydro-sarcotrocheliol **281** [90].

From *S. ehrenbergi* eight cembranoids, sarcophytonoxides A–E **282**–**286** were identified. Sarcophytonoxide A **282**, a cembrane diterpene with epoxide, dihydrofuran, acetyl group and three olefin bonds were confirmed by spectral data analysis while sarcophytonoxide D **285** was the deacetylated form of sarcophytonoxide C **284** which has a structure similar to sarcophytonoxide A **282**. However, sarcophytonoxide C **283** differed in the chemical shift of C-19, C-6, C-7, and C-9 because of the 7,8-double bond configuration or chiral center of C-6. However, sarcophytonoxide E **286** differed in the position of acetyl group and the exocyclic double bond. [91]. From *S. trocheliophorum* a sarsolenane diterpene, secodihydrosarsolenone **287** was identified [92].

The chemical investigation of both diethyl ether and dichloromethane extracts of *S. stellatum* yielded the isolation of three cembranoid diterpenes and enantiomer, (+)-(1E,3E,11E)-7,8-epoxycembra-1,3,11,15-tetraene **288**, (+)-(7*R*,8*R*,14*S*,1*Z*,3*E*,11*E*)-14-acetoxy-7,8-epoxycembra-1,3,11-triene **289** [93].

Five isoprenoids from *S. glaucum*, 3,4,8,16-tetra-epi-lobocrasol, 1,15β-epoxy-deoxysarcophine, 3,4-dihydro-4α,7β,8α-trihydroxy-∆^2^-sarcophine, ent-sarcophyolide E **290**–**293**, together with, 3,4-dihydro-4α-hydroxy-∆^2^-sarcophine, 3,4-dihydro-4β-hydroxy-∆^2^-sarcophine **294** and **295** and klyflaccicembranol F **296** were reported and their structures were elucidated by spectral data. [70]. Moreover, five cembranoids, sarelengans C–G **297**–**301** from *S. elegans* were also stated. Isolates structures were established by spectral data, and absolute configuration of sarelengans D–F **298**–**300** were investigated through single crystal X-ray diffraction [94].

Isolation of seven diterpenes were reported from *S. ehrenbergi* and identified as sarcoehrenbergilids A–C **302**–**304** together with sinulolides A and B **305** and **306**. The absolute configuration of sarcoehrenbergilid A **301** was investigated by scattering of CaKα radiation with the flack parameter [95]. Moreover, sarcoehrenbergilid D–F **307**–**309**, diterpenes isolated from *S. ehrenbergi* were isolated and their absolute configurations were investigated by experimental and TDDFT-simulated ECD spectra. Sarcoehrenbergilid D **307** was found to differ from compound **301** only in stereochemistry [96]. Furthermore, five cembranes diterpenes, Sarcoehrenolides A–E **310**–**314** were isolated from *S. ehrenbergi*. Their chemical structures were determined through extensive spectral data. All isolates were related to ehrenbergol D **251** in structure, having an α,β-unsaturated-γlactone group at carbon-6 to carbon-19, however, they differ in migration of double bonds and/or oxidative configurations. Additionally, the absolute configuration of sarcoehrenolide A **310** was investigated by a single-crystal X-ray diffraction assay by Cu Kα radiation, and the absolute configurations of sarcoehrenolides B **311** and D **313** by TDDFT/ECD calculations [97].

From *S. infundibuliforme* two nitrogenous diterpenoids with unusual tricycle [6.3.1.01,5] dodecane skeleton named, sarinfacetamides A and B **315** and **316** and a known compound; nanolobatin B **317** were reported. Their structures were clarified by a thorough spectral data, TDDFT-ECD calculation and the absolute configuration of sarinfacetamide A **315** was investigated. The authors proposed a probable biosynthetic pathway for sarinfacetamides A and B **315** and **316**, in which, the development of the carbon-12−carbon-4 bond together with epoxide ring opening of nanolobatin B **317** created an intermediary carbon cation molecule which reacted with the nitrogen lone pair electrons attacking carbon-9 followed by the opening of carbon-1/carbon-9 bond and generation of carbon-1/carbon-8 bond offering sarinfacetamides skeleton, of which acetylation of carbon -4/carbon -8 or carbon -4/carbon -8/carbon -16 yielded sarinfacetamides B **316** and A **315**, respectively [98]. From genus *sarcophyton*, (1*S*,2*E*,4*R*,6*E*,8*S*,11*S*,12*S*)-11,12-epoxy-8-hydroperoxy-4-hydroxy-2,6-cembradiene **318** was reported. Its structure was fully determined through a complete spectroscopic analysis [99].

**Figure 4:**
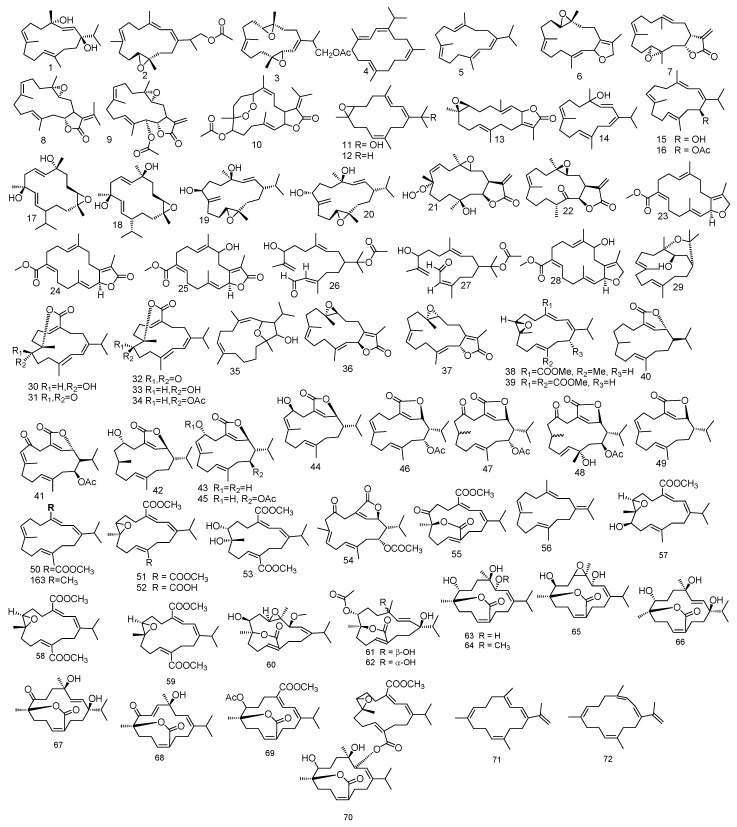

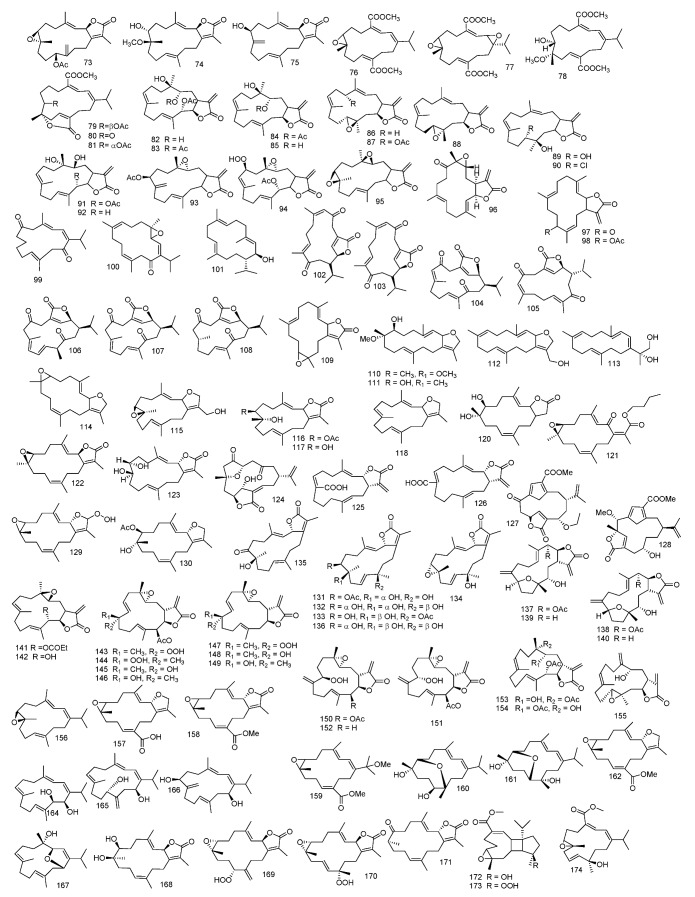

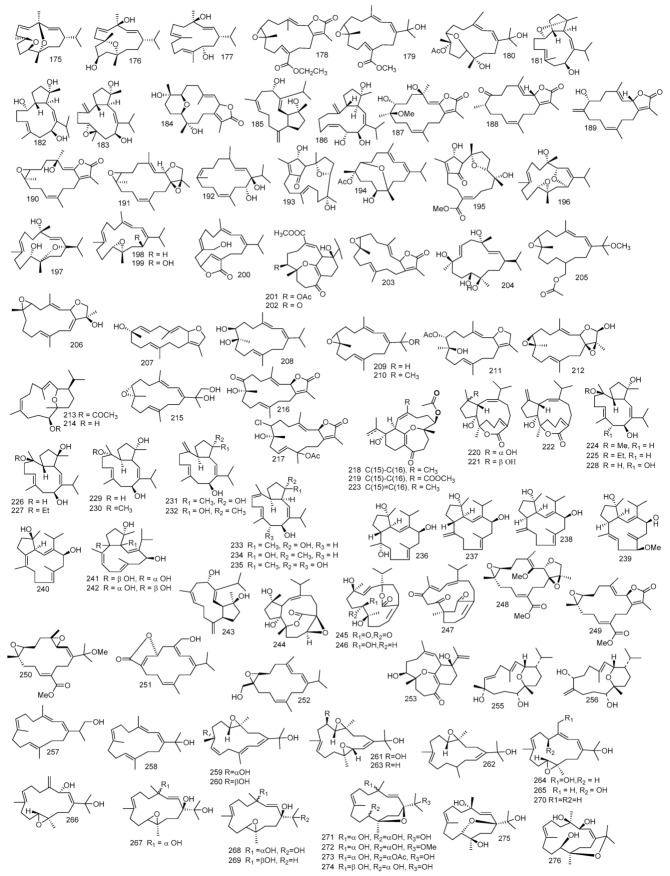

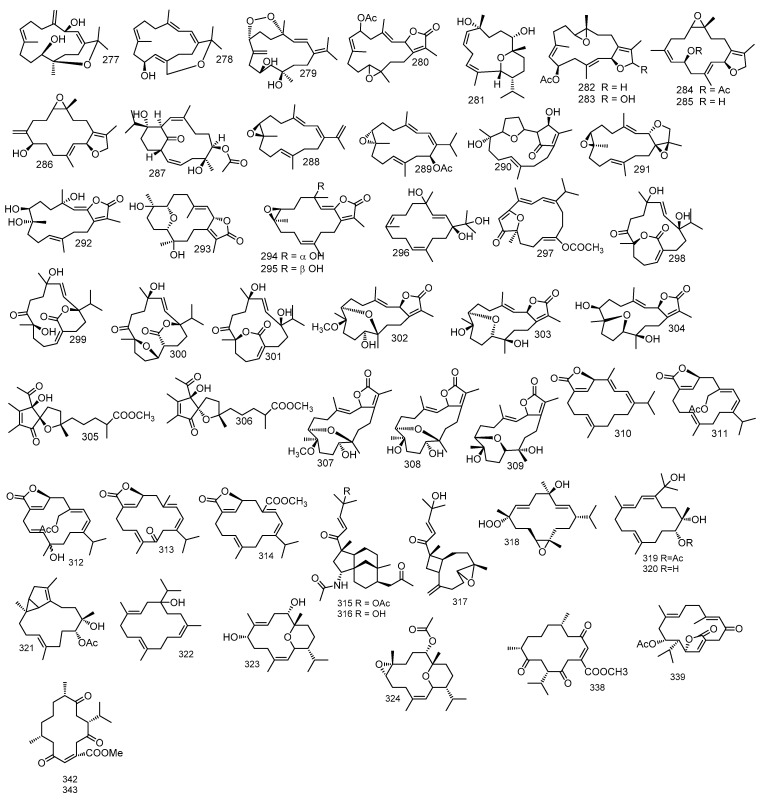
Diterpenes reported from *Sarcophyton* sp.

Sarcomililatols A, B and sarcomililate A **319**–**321**, which possessed tricyclo [11.3.0.02,16] hexadecane skeleton, along with diterpenoids sarcophytol M **322**, were isolated from *S. mililatensis*. Absolute configuration for sarcomililatol A **319** and sarcomililate A **321** were elucidated by combination of residual dipolar coupling-based NMR analysis, Snatzke’s assay and TDDFT-ECD calculation and anomalous X-ray diffraction with sarcomililatol A **319**. The authors also proposed a biogenetic pathway relationship for sarcomililatols A, B and sarcomililate A **319**–**321.** Based on structural resemblance between the three compounds, acetylation of sarcomililatol B **320** gave sarcomililatol A **319**, and with dehydration under acid, isomerization and intramolecular [4 + 2] cycloaddition, sarcomililate A **321** was formed [100]. A pyrane-cembranoid diterpenes, 9-hydroxy-7,8dehydro-sarcotrocheliol and 8,9-expoy-sarcotrocheliol acetate **323** and **324** were isolated from *S. trocheliophorum* [101]. Figure 4 summarizes diterpenes isolated from *Sacrophyton* sp.

### 2.2. Biscembranes

Four biscembranes, bisglaucumlides A–D **325**–**328** were isolated from *S. glaucum*. Spectral data showed that bisglaucumlide A **325** possessed a biscembranoid skeleton. Bisglaucumlide B **326** was confirmed to be 32-acetylbisglaucumlide A by the positive Cotton effect in the CD spectrum. As for bisglaucumlide C **327** it was found to be the geometrical isomer of bisglaucumlide B **326** while considering the geometry of the C-4 olefin. Bisglaucumlide D **328** was an isomer to bisglaucumlide C **327**, its absolute configuration indicated an anticlockwise relation among the enone chromopores revealing a negative Cotton effect CD spectrum [102]. Moreover, chemical investigation of *S. glaucum* extract yielded two biscembranes with an uncommon α, β-unsaturated ε-lactone, Glaucumolides A and B **329**–**330** [103].

Ximaolides A–G **331**–**337**, seven biscembranoid, together with methyl tortuosoate A **338** where isolated from *S. tortuosum*. Their structures were elucidated through spectral analysis and Ximaolide A **331** and E **335** relative stereochemistry were investigated using X-ray diffraction method. The authors demonstrated that methyl tortuosoate A **338** could be the biogenetic precursor for all isolated metabolites since their upper parts were closely related to compound **338** [104].

A cembranolide diterpene identified as isosarcophytonolide D **339**, an isomer to the previously isolated compound **41** from *S. tortuosum*, along with two biscembranes, bislatumlides A and B **340**–**341**, were isolated from *S. latum*. A detailed spectral analysis revealed that the structure of bislatumlide B **341** matched that of bislatumlide A **340**. However, ^13^C NMR data revealed a significant difference from compound **340** in the chemical shifts of carbon-19 and carbon-10 demonstrating the Z nature of Δ^11^ olefin in compound **340**. Thus, compound **340** was found to be the 11Z isomer of bislatumlide B **341**. Interestingly the authors have proposed a biosynthetic pathway for bislatumlides A and B **340**–**341** in which isosarcophytonolide D **339** was found to be one of the precursors for bislatumlide A **340**. Moreover, the authors investigated the effect of long-term storage in CDCl_3_, where it showed isomerization of bislatumlide A **340** to bislatumlide B **341** at ∆^11^ [105].

Methyl tetrahydrosarcoate and methyl tetrahydroisosarcoate **342** and **343**, two cembranoids isolated from *S. elegans*, along with four biscembranoids, nyalolide, desacetylnyalolide, diepoxynyalolide, and dioxanyalolide **344**–**347**. The authors proposed that diepoxynyalolide **346** could be a precursor for both compound nyalolide **344** and dioxanyalolide **347** [106].

Investigation on *S. elegans* extract led to the isolation of six biscembranoids identified as sarcophytolides G–L **348**–**353**, together with biscembranoids, lobophytones H, Q, K, W, U **354**–**358**. Isolates structure were determined by spectroscopic analysis. Absolute configuration of the compound sarcophytolide G **348** was determined using Mosher reaction [22,107]. From the methanol extract of *S. pauciplicatum*, sarcophytolides M and N **359** and **360**, along with lobophytone O **361**, were isolated [108].

**Figure 5:**
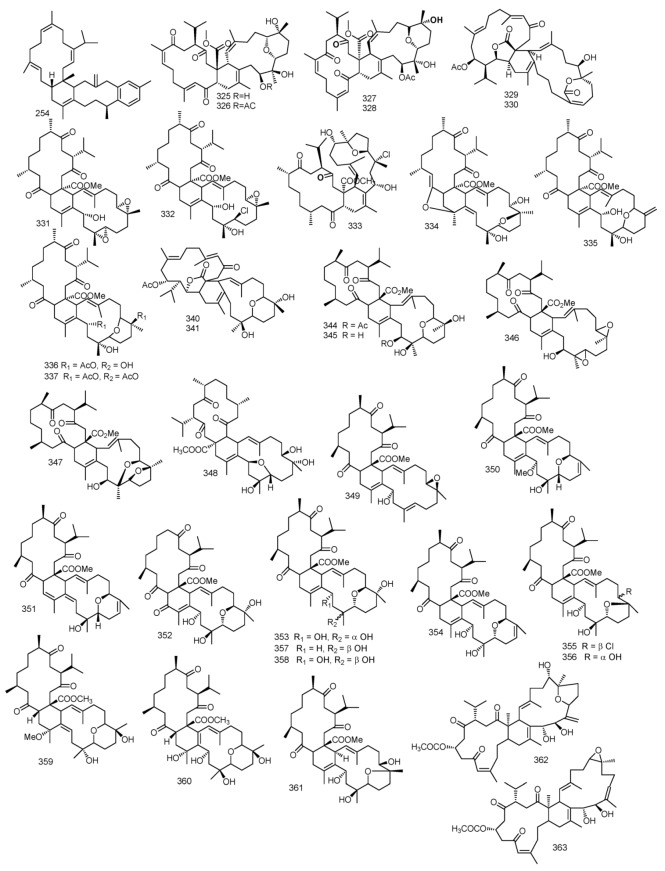
Biscembranes reported from *Sarcophyton* sp.

Two biscembranoids, sarelengans A and B **362** and **363**, were reported from *S. elegans*. Their chemical structures were investigated by spectral and chemical methods, and the absolute configuration of sarelengans A determined by single crystal X-ray diffraction. Sarelengans A and B **362** and **363** possessed a conjuncted trans-fused A/B-ring between two cembranoid entities. The authors mentioned that this structure feature led to an uncommon biosynthetic pathway including a cembranoid-∆^8^ instead of cembranoid-∆^1^ unit in endo-Diels-Alder cycloaddition [94]. Figure 5 summarizes biscembranes isolated from *Sacrophyton* sp.

### 2.3. Sesquiterpenes

Investigation of the methylene chloride extract of *S. acutangulum* yielded tetracyclic terpenoid hydrocarbon (+)-alloaromadendrene **364** which showed similar spectral data as that of (−)-alloaromadendrene but with different optical rotation [R]D +25.8° (−)-alloaromadendrene and cyclosinularane **365** [109].

Two guaiane sesquiterpenes 4α-ethoxy-10α-hydroxyguai-6-ene and 10α-hydroxy-4α-methoxyguai-6-ene **366** and **367** were isolated from *S. buitendijki* and their structures were elucidated through 1 and 2D NMR [110]. One 1,2-dioxolane sesquiterpene alcohol named, dioxosarcoguaiacol **368**, was isolated from *S. glaucum* [111].

**Figure 6:**
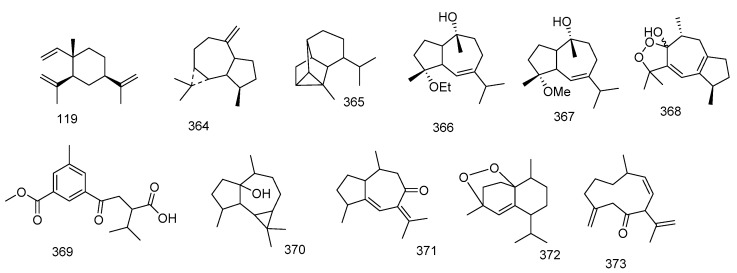
Sesquiterpenes reported from *Sarcophyton* sp.

Trocheliophorin **369** was isolated from *S. trocheliophorum* ethyl acetate extract. Through spectral data, its structure was elucidated, revealing that it could be the result of aromatization with dehydration of ring B of sarcophytin which co-exist in the extract, and removal of ring C and the ring junction methyl and breakage of ring A [112]. In addition, aromadendrene sesquiterpenoid, palustrol **370** from *S. trocheliophorum* was reported [77]. Moreover, sesquiterpene guajacophine **371** and 1,4-peroxymuurol-5-ene **372** from *S. ehrenbergi* were stated. [62]. Continuing the abovementioned isolation from *S. glaucum* sesquiterpenoid, 6-oxo-germacra-4(15),8,11-triene **373** was also reported [78]. Figure 6 summarizes sesquiterpenes isolated from *Sacrophyton* sp.

### 2.4. Polyhydroxysterol and Steroids

One polyhydroxysetrol, 23,24-dimethylcholest-16(17)-*E*-en-3β,5α,6β,20(*S*)-tetraol **374**, along with 24-methylcholestane-3β,5α,6β,25-tetraol-25-monoacetate **375** and gorosten-5(*E*)-3β-ol **376**, were reported from *S. trocheliophorum*. Interpretation using 1 and 2D NMR analysis pointed out the existence of 23,24-dimethyl cholesterol derivatives which were further approved by the mass fragmentation pattern [29]. The isolation of (24*S*)-24methylcholestane-3β,5α,6β-triol **377** from *S. crassocaule* were also reported [28].

Sardisterol **378** was isolated from *S. digitatun* Moser. The carbon NMR matched that of (22*R*)-methylcholest-5-en-3β, 22,25,28-tetraol-3,22,28-triacetate **379** indicating that sardisterol **378** has the same steroidal nucleus as (22*R*) -methylcholest-5-en-3β, 22,25,28-tetraol-3,22,28-triacetate **378** but the OH groups in carbon 22 and 28 were replaced by acetoxy groups [113].

(24*S*)-24-methylcholestane-3β,5α,6β,25γ,26-pentol-25,26-diacetate **380** and (24*S*)-24-methylcholestane-3β,5α,6β,25γ,26-pentol-26-*n*-decanoate **381**, was isolated from *S. trocheliophorum*, while, (24*S*)-24-methylcholestane-3β,5α,6β,25γ-tetrol **382** and (24*S*)-24- methylcholestane-3β,5α,6β,25γ pentol-25-monoacetate **383** were reported from *S. glaucum* [114].

Fourteen polyoxygenated steroids with 3β,5α,6β-hydroxy group, showing ergostane, cholestane, gorgostane and 23,24-dimethyl cholestane carbon skeletons were reported from *Sarcophyton* sp., 11α-acetoxy-cholesta-24-en-3β,5α,6β-triol **384**, (22*E*,24*S*)-11α-acetoxy-ergostane-22,25-dien-3β,5α,6β-triol **385**, (24*S*)-ergostane-1α,3β,5α,6β,11α-pentaol **386**, (24*S*)-23,24-dimethylcholesta-22-en-3β,5α,6β,11α-tetraol **387**, (23*R*,24*R*)-23,24-dimethylcholesta-17(20)-en-3β,5α,6β-triol **388**, 11α-acetoxy-gorgostane-3β,5α,6β,12α-tetraol **389** and 12α-acetoxy-gorgostane-3β,5α,6β,11α-tetraol **390**, sarcoaldosterol A **391**, (24*S*)-ergostane-3β,5α,6β-triol **392**, (24*S*)-ergostane-3β,5α,6β,11α-tetraol **393**, (24*S*)-ergostane-7-en-3β,5α,6β-triol **394**, 11α-acetoxy-gorgostane-3β,5α,6β-triol **395**, sarcoaldosterol B **396** and gorgostane-1α,3β,5α,6β,11α-pentaol **397**. Structural elucidation for all isolates were done based on spectral analysis and comparing with reported literature [115].

Six polyhydroxy steroids, (24*S*)-ergostan-3β,5α,6β,25-tetraol-25-monoacetate **398**, (24*S*)-24-methylcholestan-3β,6β,25-triol-25-O-acetate **399**, (24*S*)-methylcholestan-3β,5α,6β,25-tetraol-3,25-diacetate **400**, (24*S*)-24-methylcholestan-1β,3β,5α,6β,25-pentaol-25-monoacetate **401** and (24*S*)-methylcholestan-3β,5α,6β,12β,25-pentaol-25-*O*-acetate **402**, were reported from *Sarcophyton* sp., one was reported as 18-oxygenated polyhydroxy steroid, (24*S*)-ergostan-3β,5α,6β,18,25-pentaol 18,25-diacetate **403**. The structure of this compound was determined through spectroscopic data, and its absolute configuration was elucidated by the modified Mosher’s assay [116].

Chemical investigation of the polar fraction of *S. trocheliophorum*, yielded two poly-hydroxy steroids, identified through extensive spectral analysis as zahramycins A and B **404** and **405**. Zahramycin A **404** was characterized by the existence of oxirane ring at carbon-5 and carbon-6, while zahramycin B **405** possessed a keto-hydroxy sterol structure [117].

Ten polyhydroxylated steroids were isolated from *Sarcophyton* sp., (23*R*,24*R*,17*Z*)-11α-acetoxy-16β-methoxy-23,24-dimethylcholest-17(20)-en-3β,5α,6β-triol **406**, (24*R*)-gorgost-25-en-3β,5α,6β,11α-tetraol **407** and 11α-acetoxycholest-24-en-1α,3β,5α,6β-tetraol **408**, (24*R*)-methylcholest-7-en-3β,5α,6β-triol **409**, 11α-acetoxy-cholest-24-en-3β,5α,6β-triol **410,** (22*E*,24*S*)-11α-acetoxy-ergost-22,25-dien-3β,5α,6β-triol **411**, (24*S*)-11α-acetoxy-ergost-3β,5α,6β-triol **412**, (24*R*)-11α-acetoxy-gorgost-3β,5α,6β-triol **413**, (24*S*)-ergost-3β,5α,6β,11α-tetraol **414**, and (24*S*)-23,24-dimethylcholest-22-en-3β,5α,6β,11α-tetraol **415**. Their structural elucidation was based on spectral data, and it was found that all isolated compounds have a distinguishable 3β,5α,6β-trihydroxy group; however, they differ in side chains and substitutions. These steroids could be alienated structurally into four categories including, cholesterol, ergosterol, gorgosterol and 23,24-dimethyl cholesterol. (23*R*,24*R*,17*Z*)-11α-acetoxy-16β-methoxy-23,24-dimethylcholest-17(20)-en-3β,5α,6β-triol **406** has a distinctive 17(20)-en-23,24-dimethyl side chain, while (24*R*)-gorgost-25-en-3β,5α,6β,11α-tetraol **407** was a gorgosterol having a 25-ene side chain [118].

Ethanol-soluble fraction of the acetone extract of *S. trocheliophorum* yielded 9,11-secosteroid named, 25(26)-dehydrosarcomilasterol **416** and three polyhydroxylated steroids, 7α- hydrocrassorosterol A **417**, 11α-acetoxy-7α-Hydrocrassorosterol A **418**, sarcomilasterol **419**, 3β,6α,11-trihydroxy-9,11-seco-5α-cholest-7-ene-9-one **420** and 3β,6α,11-trihydroxy-24-methylene-9,11-seco-5α-cholest-7-ene-9-one **421**. The 9,11-secostroids nucleus can be described as the chemotaxonomic indicators for genus *Sarcophyton* [119].

**Figure 7:**
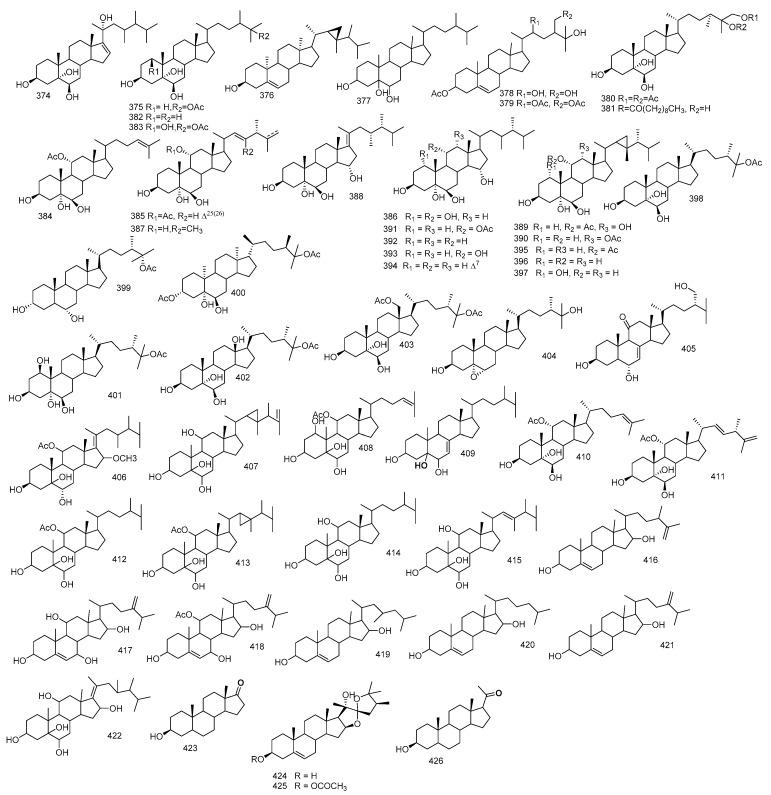
Polyhydroxylated sterols reported from *Sarcophyton* sp.

Beside the abovementioned isoprenoids obtained from *S. glaucum*, 16-deacetylhalicrasterol B **422**, together with sarcoaldesterol B **396**, sarglaucsterol **423** were isolated too and their structures were elucidated by spectral data [70]. Furthermore, from *S. ehrenbergi* the isolation of two formerly isolated hippurine **424** and **425** [120] alongside pregnenolone **426** were reported [121]. Figure 7 summarizes polyhydroxylated sterols isolated from *Sacrophyton sp*.

### 2.5. Miscellaneous

From *S. trocheliophorum*, tetradecyl octadecenoate **427**, 2,3-dihydroxypropyloctadecyl ether **428** and tetradecyl-9-*Z*-octadecenoate **429** were identified [29]. In addition, purification of the total lipid extract of *S. trocheliophorum* provided four butenolides **430**–**433** with different chain substitutions and saturation together with three fatty acids, arachidonic acid, eicosapentaenoic and docosahexaenoic methyl esters **434**–**436** and prostaglandin PGB2 **437** [122].

An infrequent prostaglandin was isolated from *S. crassocaule*, (5*Z*)-9,15-dioxoprosta-5,8(12)-dien-1-oate **438** based on spectral analysis. This was the first time to report a prostaglandin with a C-15 keto group from natural origin [123]. Furthermore, from the ethyl acetate and n-butanol fractions of *S. crassocaule*, two isolated metabolites identified as sarcophytonone **439** a tetra-substituted quinone, and sarcophytonamine **440** a quaternary amine were reported. It might be valuable to know that these quinone derivatives are scarce in marine organisms and only sarcophytonone **439** was identified in *S. mayi* [124].

Five compounds were isolated from *S. infundibuliforme*, three were reported *O*-glycosylglycerol known as sarcoglycosides A–C **441**–**443** and chimyl alcohol and hexadecanol **444** and **445**. Sarcoglycoside A **441** was the first glycoglycerolipid to be isolated from soft coral, while sarcoglycosides B and C **442** and **443** were rare marine isolates, composed of a lyxose residue and chimyl alcohol moiety [125]. Moreover, one α-tocopheryl quinone derivative, 3,5,6-trimethyl-2-14*S*-3,11,14-trihydroxy-3,7,11,15-tetramethylhexadecylcyclohexa-2,5-diene-1,4-dione **446**, was isolated [64].

Purification of ethyl acetate extract of *S. ehrenbergi* yielded ten prostaglandins, sarcoehrendin A−J **447**–**456** together with five correlated compounds **457**–**461**. Sarcoehrendin A **447** was found to be the acetylated derivative of arachidonic acid ethyl; previously isolated from *Lobophyton depressum* [126,127]. Another six prostaglandins **462**–**467**, were reported from *S. ehrenbergi*, three were reported to be of marine origin [121]. From *S. ehrenbergi* extract, 2-methyl-1-octanol ester of (*E*)-3-(4methoxyphenyl) propenoic acid **468** was reported. The authors mentioned that stereochemical structure of 2-methyl-1-octanol ester of (*E*)-3-(4methoxyphenyl) propenoic acid **468** was ensured via synthesis of two possible isomers (*S*)-1 and (*R*)-1 which was recognized by an asymmetric synthesis using 4-benzyl-2-oxazolidinone chiral auxiliaries from octanoic acid [128]. From *S. ehrenbergi* ceramide **469** was reported alongside two cerebrosides, sarcoehrenosides A and B **470** and **471**. A detailed spectral analysis revealed the occurrence of an amide linkage, a long chain, and a sugar, dependable with the C-9 methyl cerebroside nature of sarcoehrenoside A **470** [129].

Three carotenoids, peridinin, peridininol and peridininol-5,8-furanoxide **472**–**474** were reported for the first time from *S. elegans*. Chemical structures were interpreted by using spectral data and reported data [130]. Additionally, from *Sarcophyton* sp. another carotenoid, all-trans-(9′*Z*,11′*Z*)-(3*R*,3′*S*,5′*R*,6′*R*)-pyrrhoxanthin **475** was isolated [76].

**Figure 8:**
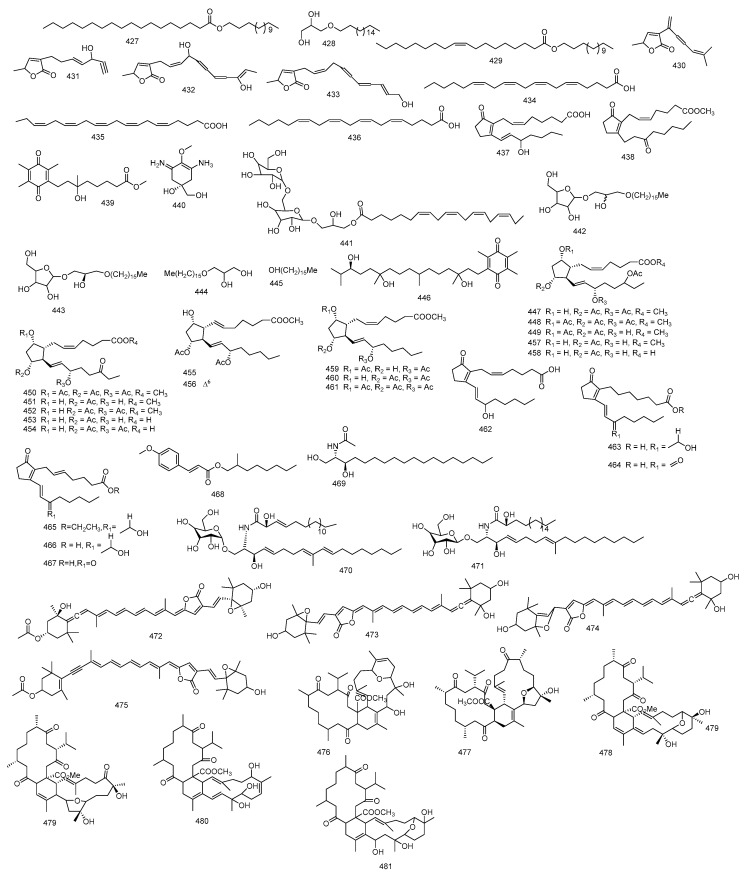
Miscellaneous isolated from *Sacrophyton* sp.

Methyl tortuoate A and methyl tortuoate B **476** and **477**, two tetracyclic tetraterpenoids, together with methyl sartortuoate **478** and methyl isosartortuoate **479**, were reported from *S. tortuosum*. Methyl tortuoate A **476** was similar to methyl sartortuoate **478** in structure, except for the presence of secondary hydroxyl group in methyl tortuoate A **476** and absence of one tertiary hydroxyl functional group and conjugated diene. As for, methyl tortuoate B **477,** it was found to be similar to methyl isosartortuoate **479** in structure, but with no hydroxyl group at C-27 [131]. Tetraterpenoid, methyl tortuoate C **480** after further investigation of the same ethanolic extract of *S. tortuosum* was isolated and a full spectral data was done to investigate its structure [132]. Another tetracyclic tetraterpenoid; methyl tortuoate D **481**, was also reported from *S. tortuosum* and was identified using direct infusion electrospray ionization mass spectrometry [133]. Figure 8 summarizes miscellaneous isolated from *Sacrophyton* sp.

## 3. Biological Activities

### 3.1. Cytotoxic Activity

The capability of 13- Acetoxysarcocrassolide **9** was investigated, as a cytotoxic agent against gastric carcinoma using MTT method, colony formation method, cell morphology assessments, and wound-healing method. It suppressed the development and migration of gastric cancer cells in a dose-dependent manner and initiated both early and late cell death examined by flow cytometer assay [134]. The authors mentioned that there was a relationship between the structure of sarcrassin A, B, D, and E **76**, **77**, **79**, and **80**, and emblide **81**, and its activity, showing that loss of acetoxy group as in crassocolide C **84** led to loss of activity against all tested cell lines. While, acetylation at 4-OH position in crassocolide B **83** resulted in a decrease in activity cytotoxicity. However, the existence of two hydroxy moiety present at carbon-3 and carbon-4 and no oxidation at carbon-13 as in crassocolide D **85** showed potent activity against MCF-7 and A549 cell lines. While, crassocolide A **82** and F **87** exhibited potent activity toward Hep G2, MCF-7, MDA-MB-231 and A549, because of the 5-*O*-acetyl group [46]. Furthermore, crassocolide H and L **90** and **94**, from *S. crassocaule*, showed strong activity toward KB, Hela, and Daoy cell lines owing to the presence of Cl atom at C-11 instead of OH group in crassocolide H **90** [47].

Sarcocrassocolides A–D **137**–**140**, showed potent activity toward MCF-7, WiDr, HEp-2 and Daoy cell lines [58]. The authors maintained that the existence of acetoxy group at C-13 was important for activity. Sarcocrassocolides F–I **143**–**146**, showed cytotoxicity toward all or part cell lines. However, sarcocrassocolide I **146** was most potent toward Daoy, HEp-2, MCF-7 and WiDr cell lines while sarcocrassocolide J and L **147** and **149** 13-deacetoxy derivatives, were least potent against all tested cell lines with ED_50_ = >20 μM. Furthermore, hydroxy moiety at carbon-8 improve the cytotoxic activity in contrast with carbon-8 hydroperoxy-bearing correspondents sarcocrassocolide F and H **143** and **145** were most potent toward MCF-7 [59].

Owing to the α,β-unsaturated ε-lactone ring in glaucumolides A and B **329** and **330** both exhibited strong cytotoxicity toward HL-60 and CCRF-CEM cell [103]. The authors specified that the less degrees of oxidation the more immunosuppressive activity, yalongene A **71** was the most potent even better than the positive control Cyclosporin A [100]. (24*S*)-24-methylcholestane-3β,5α,6β,25-tetrol-25-monoacetate **375** exhibited potent activity toward P-388, A549, and HT-29 cell lines [114]. The authors reported that there was a structure activity relationship in which the existence of an extra free hydroxyl group at C-20 position in 23,24-dimethylcholest-16(17)-*E*-ene-3β,5α,6β,20(*S*)-tetraol **374**, and acetyl group at C-25 position in 24-methylcholestane-3β,5α,6β,25-tetraol 25-monoacetate **375** led to strong cytotoxicity toward human M14, HL60, and MCF7 cells with a dose-dependent manner [29]. The occurrence of OAc moiety at carbon-11 was important for cytotoxic activity, as in (23*R*,24*R*,17*Z*)-11α-acetoxy-16β-methoxy-23,24-dimethylcholest-17(20)-en-3β,5α,6β-triol **406**, (22*E*,24*S*)-11α-acetoxy-ergost-22, 25-dien-3β,5α,6β-triol **411** and (24*R*)-11α-acetoxy-gorgost-3β,5α,6β-triol **413** showed a strong cytotoxicity toward K562, HL-60, HeLa cell lines, while, 11α-acetoxycholest-24-en-1α,3β,5α,6β-tetraol **408**, 11α-acetoxy-cholest-24-en-3β,5α,6β-triol **410** and (24*S*)-11α-acetoxy-ergost-3β,5α,6β-triol **412** exhibited a potent activity toward K562 and HL-60 [118].

### 3.2. Anti-Inflammatory Activity

Sarcocrassocolide M **150** could be a leading anti-inflammatory. Sarcocrassocolides M–O **150**–**152** might be beneficial anti-inflammatory agents because of the structure relationship and the existence of β-hydroperoxy moiety at carbon-7 [60]. Sarcocrassocolides F–L **137**–**143** activity was attributed to the ring-opening of the α,β-unsaturated-β-ether ketone group leading to an increase in the enzyme inhibitory activity [58]. Sarcoehrenolide A, B, and D **310**, **311**, and **313** and ehrenbergol D **251** showed significant TNF-α inhibition in which sarcoehrenolide B **311** was most active due to the existence of acetoxy at carbon-18. A structure activity relationship was demonstrated in which the keto moiety at carbon-13 and hydroxyl group at carbon-18 could be responsible for the slight increase in activity. However, the presence of carbomethoxy moiety at carbon-18 led to a reduction in activity [97].

### 3.3. Antidiabetic Activity

Methyl sarcotroate B **173** has strong inhibitory activity toward PTP1B because of the hydroperoxide group which binds to the active site of the Cys residue [67]. Potency of sarcophytonolide N **50** and sarcrassin E **80** may be because of the existence of methyl ester moiety at carbon-18, which significantly increases the enzyme inhibitory activity toward human PTP1B enzyme [39].

### 3.4. Antimicrobial Activity

Sarcophytolide **32** showed a strong antibacterial activity toward methicillin-sensitive *S. aureus* Newman strain because of the diene at C-1/C-3 [41]. The crude extract exhibited antimicrobial activity toward most of the examined bacteria, yeasts, and fungi. [77]. Trocheliophols H, I, L, N, O, and R **231**, **232**, **235**, **237**, **238**, and **241**, 4-epi-sarcophytol L **243** showed antibacterial activity toward *Xanthomonas vesicatoria*, *Agrobacterium tumefaciens*, *Pseudomonas lachrymans*, *Bacillus subtilis*, and *Staphylococcus aureus*. The authors mentioned that the structure activity relationship and the existence of exomethylene group at C-8 add to the antibacterial activity, while H-3β orientation, which was present only in compound trocheliophol S **242**, gave the most potent activity against the selected bacteria [81]. The toxicity of the novel γ-lactones compounds butenolides **430**–**433** were evaluated by using shrimp bioassay, and bioactivity was shown. Additionally, they showed activity against Gram-positive bacteria only [122].

Because of the structure activity relationship, 11α-acetoxy-cholesta-24-en-3β,5α,6β-triol **384**, (22*E*,24*S*)-11α-acetoxy-ergostane-22,25-dien-3β,5α,6β-triol **385**, 11α-acetoxy-gorgostane-3β,5α,6β,12α-tetraol **389**, 12α-acetoxy-gorgostane-3β,5α,6β,11α-tetraol **390**, and sarcoaldosterol A **391** were more potent toward antibacterial activity toward *Escherichia coli* and *Bacillus megaterium*, and antifungal activity toward *Microbotryum violaceum* and *Septoria tritici* fungi, because of the 11*α*-acetoxy group, cyclopropane side chain and terminal-double bond [115].

### 3.5. Miscellaneous

Anticonvulsant activity of ceramide **469**, measured in vivo by the pentylenetetrazole (PTZ)-induced seizure assay, has successfully opposed the lethality of pentylenetetrazole in mice. It showed also a significant anxiolytic activity when used in the light–dark transition box. This could be caused possibly by GABA and serotonin receptors modulation [135]**.** Table 1 summarizes the main biological activities of secondary metabolites from genus *Sacrophyton*.

## 4. Conclusions

Based on reviewing the available current literature, a huge library of metabolites was isolated, and it possessed unique structures. Up to 481 compounds with different structures belonging to different chemical classes were reported from the *Sarcophyton* species. The chemical structures were classified as terpenoids (majority), biscembranes, polyhydroxylated sterols, sesquiterpenes (minority), and miscellaneous compounds. *S. trocheliophorum* gave the highest number of compounds. Members of genus *Sarcophyton* possessed valuable and interesting biological activities, such as antibacterial, cytotoxicity, antifungal, and antidiabetic.

## Figures and Tables

**Table 1:** The main biological activities of secondary metabolites isolated from genus *Sacrophyton*.

Compound Name (Number)	Soft Coral	Chemical Class	Biological Activities	Geographical Area of Collection
Crassolide **7**	*S. crassocaule*	Diterpene	Potent cytotoxic activity against A549, HT-29, KB with IC_50_ range of 7.55 to 9.15 and most active against P-388 cell line with ED_50_ = 0.16 µg/mL [28].	Green Island, Taiwan
Sarcocrassolide A **8**			Potent cytotoxic activity against A549, HT-29, KB with IC_50_ range of 4.29 to 8.35 and most active against P-388 cell line with ED_50_ = 0.14 µg/mL. Significantly decreased iNOS protein levels and COX-2 expression to 1.1 ± 0.9% and 3.9 ± 2.3%, respectively, could be a promising anti-inflammatory agent [32,58].	Green Island, Taiwan. Xisha Islands, South Sea, China. Dongsha coast, Taiwan
13-Acetoxysarcocrassolide **9**			Potent cytotoxic activity against A549, HT-29, KB with IC_50_ range of 4.66 to 7.39 and most active against P-388 cell line with ED_50_ = 0.38 µg/mL and gastric carcinoma [32,58].	Green Island, Taiwan
Denticulatolide **10**	*S. crassocaule Sarcophyton* sp.		Potent cytotoxic activity against A549, HT-29, KB with IC_50_ range of 5.78 to 6.46 and most active against P-388 cell line with ED_50_ = 0.15 µg/mL. Inhibited the colony formation of Chinese hamster V79 at ED_50_ = 3.6 µM, respectively and decreased the TNFα-production at 3.0–10.0 µM [28,54].	Green Island, Taiwan. Manado, North Sulawesi
Sarcophin **13**	*S. glaucum* *S. elegans* *S. mililatensis*		Significantly decrease the viability of melanoma cells and does not show toxic effect on CV-1 cells and decrease de novo DNA synthesis and PARP activity. Exhibited cytotoxic activity toward A2780 cell line with IC_50_ > 10 μg/mL. Significant increase in ALP activity and collagen synthesis [75,136].	Xidao Island, Hainan, China Baycanh Island, Condao District, Baria-Vungtau province, Vietnam
Sarcophytol A **15**	*S. infundibuliforme*		Strong antifouling activity toward the larval settlement of barnacle *Balanus Amphitrite* (EC_50_ = 2.25 µg/mL) [64].	Wenchang coral reef in the South China Sea
Sarcophytol A acetate **16**			Strong antifouling activity toward the larval settlement of barnacle *Balanus Amphitrite* (EC_50_ = 1.75 µg/mL) [64].	
13-Dehydroxysarcoglaucol **23**	*S. cherbonnieri*	Diterpene	Potent cytotoxic activity against hepatocellular carcinoma, gastric adenocarcinoma, and breast adenocarcinoma cell lines against cell lines with IC_50_ = 6.6, 5.4, 1.7 μg/mL, respectively [33].	Ra-Ra Reef, Fiji Islands, and Stanley Reef, Australia
Sarcoglaucol-16-one **25**	*S. cherbonnieri* *S. ehrenbergi*		Potent cytotoxic activity against hepatocellular carcinoma, gastric adenocarcinoma, and breast adenocarcinoma cell lines against cell lines with IC_50_ = 8.6, 7.1, 6.1 μg/mL, respectively [33].	
Decaryiol **29**	*S. cherbonnieri*		Potent cytotoxic activity against hepatocellular carcinoma, gastric adenocarcinoma, and breast adenocarcinoma cell lines against cell lines with IC_50_ = 2.0, 7.1, 0.19 μg/mL, respectively [33].	
Sarcophytolide **32**	*S. glaucum* *S. trocheliophorum*		Cytotoxic activity at 500 µM concentration toward mouse melanoma B16F10 cells. Good antidiabetic activity with IC_50_ = 15.4 µM. Strong antibacterial activity toward methicillin-sensitive *S. aureus* Newman strain with MIC = 125 µg/mL [40,41,68].	Red Sea. Yalong Bay, Hainan Province, China
(4*Z*,8*S*,9*R*,12*E*,14*E*)-9-Hydroxy-1-isopropyl-8,12-dimethyloxabicyclo [9.3.2]-hexadeca-4,12,14-trien-18-one **33**	*Sarcophyton* new sp.		Potent cytotoxicity toward breast adenocarcinoma cell line IC_50_ = 6.5 μg/mL [35].	Stanley Reef and Great Barrier Reef, Australia
Sarcophytolol **35**	*S. glaucum*		Potent activity against HepG2 with IC_50_ = 20 ± 0.032 µM [34].	North of Jeddah, Saudi Arabia, Red Sea
Sarcophytolide B **36**			Potent toward MCF-7 with IC_50_ = 25.0 ± 0.160 µM [34].	
Sarcophytolide C **37**			Potent activity against HepG2 with IC_50_ = 20 ± 0.153 µM [34].	
Sarcophytonolide J **47**	*S. infundibuliforme*		Strong antifouling activity toward the larval settlement of barnacle *Balanus Amphitrite* (EC_50_ = 7.50 µg/mL) [64].	Wenchang Coral Reef in the South China Sea
Sarcophytonolide N **50**	*S. trocheliophorum*		Strong antidiabetic activity with IC_50_ = 5.95 µM [39].	Yalong Bay, Hainan Province, China
Ketoemblide **55**	*S. elegans*	Diterpene	Significant cytotoxicity toward breast cancer MDA-MB-231 migration in a time dependent manner. Mild antidiabetic activity with IC_50_ = 27.2 µM [18,39].	Xisha Islands, South China Sea. Yalong Bay, Hainan Province, China
Yalongene A **71**	*S. mililatensis*		Most potent immunosuppressant with IC_50_ = 4.8 μM and selective index = 7.2. Strong cytoprotective activity on SH-SY5Y cell injury caused by hydrogen peroxide in vitro [42,100].	Xigu Island, Hainan Province, China
Sarcrassin A **76**	*S. crassocaule*		Potent cytotoxic activity toward KB cell lines with IC_50_ = 19.0 µg/mL [44].	Bay of Sanya, Hainan Island, China. Yalong Bay, Hainan Province, China
Sarcrassin B **77**			Potent cytotoxic activity toward KB cell lines with IC_50_ = 5.0 µg/mL [44].	
Sarcrassin D **79**			Potent cytotoxicity toward KB cell lines with IC_50_ = 4.0 µg/mL [44].	
Sarcrassin E **80**	*S. crassocaule* *S. trocheliophorum*		Potent cytotoxic activity toward KB cell lines with IC_50_ = 13.0 µg/mL. Strong antidiabetic activity with IC_50_ = 6.33 µM [39].	
Emblide **81**	*S. crassocaule* *S. tortuosum*		Potent cytotoxic activity toward KB cell lines with IC_50_ = 5.0 µg/mL. Mild inhibition of the elastase release 29.2 ± 6.1% [44,74].	Sanya Bay, Hainan Island, China. Lanyu Island Coast, Taiwan
Crassocolide A **82**	*S. crassocaule*		Potent cytotoxic activity toward Hep G2, MCF-7, MDA-MB-231, A549 DLD-1, and CCRF-CEM cell lines (IC_50_ = 3.1, 8.9, 8.6, and 11.9 µg/mL, 5.7 and 6.3 µM, respectively). Strongly decreased iNOS protein levels and COX-2 expression to 3.5% ± 0.9% and 59.4% ± 21.4%, respectively [46,61].	Kenting Coast, Taiwan. Dongsha Coast, Taiwan
Crassocolide B **83**			Decrease cytotoxic activity against Liver, breast, lung, DLD-1, CCRF-CEM, and HL-60 cancer cells (IC_50_ = 13.1, 10.3, 12.1 11.9 µg/mL, 28.1, 8.7 and 11.1 µM, respectively). Strongly decreased iNOS protein levels to 3.2% ± 0.7% [46,61].	
Crassocolide D **85**	*S. crassocaule*	Diterpene	Potent cytotoxic activity toward MCF-7, A549, and DLD-1 cell lines with IC_50_ = 15.3, 12.5 µg/mL and 27.7 µM, respectively. Strongly decreased iNOS protein levels to 3.2% ± 0.6% [46,61].	Kenting Coast, Taiwan. Dongsha Coast, Taiwan
Crassocolide E **86**			Potent cytotoxicity toward DLD-1, CCRF-CEM, and HL-60 cancer cells with IC_50_ = 8.7, 7.3, and 8.4 µM, respectively. Strongly decreased iNOS protein and COX-2 expression levels to 1.4% ± 0.4% and 32.0% ± 15.3%, respectively [46,61].	Dongsha Coast, Taiwan
Crassocolide F **87**			Potent cytotoxic activity toward Hep G2, MCF-7, MDA-MB-231, and A549 with IC_50_ = 2.1, 7.4, 8.8, and 3.2 µg/mL, respectively [46].	Kenting Coast, Taiwan
Crassocolide H **90**			Strong cytotoxic activity toward KB, Hela, and Daoy cell lines with IC_50_ = 5.3, 14.9, and 3.8 20 µg/mL, respectively [47].	
Crassocolide I **91**			Potent cytotoxic activity toward Daoy cell line with IC_50_ = 0.8 µg/mL [47].	
Crassocolide J **92**			Potent cytotoxic activity toward Daoy cell line with IC_50_ = 2.8 µg/mL [47].	
Crassocolide K **93**			Potent cytotoxic activity toward Daoy cell line with IC_50_ = 2.5 µg/mL [47].	
Crassocolide L **94**			Strong cytotoxic activity toward KB, Hela, and Daoy cell lines with IC_50_ = 12.2, 8.0, and 4.1 µg/mL [47].	
Crassocolide M **95**			Potent cytotoxic activity toward Daoy cell line with IC_50_ = 1.1 µg/mL [47].	
Crassocolide N **96**			Potent cytotoxic activity against KB, HeLa, and Daoy cells (IC_50_ = 4.7, 4.7, and 2.8 µg/mL, respectively) [47].	Dongsha Atoll, Taiwan
Crassocolide O **97**			Potent cytotoxicity against Daoy cells IC_50_ = 4.5 µg/mL [47].	
Crassocolide P **98**	*S. crassocaule*	Diterpene	Potent and selective cytotoxicity against Daoy cells growth IC_50_ = 1.9 µg/mL [47].	Dongsha Atoll, Taiwan
Sarcostolide A **102**	*S. stolidotum*		Potent cytotoxic activity toward HeLa and WiDr cell lines with IC_50_ = 22.26 and 19.97 μg/mL, respectively [50].	Kenting, off the southern coast, Taiwan
Sarcostolide B **103**			Potent cytotoxic activity toward WiDr with IC_50_ = 8.31 μg/mL and HeLa and cell lines with IC_50_ = 5.88 μg/mL [50].	
Sarcostolide C **104**			Most potent cytotoxic activity toward HeLa cell lines with IC_50_ = 1.65 *μ*g/mL and WiDr with IC_50_ = 19.35 μg/mL [50].	
Sarcostolide D **105**			Potent cytotoxic activity toward HeLa and WiDr cell lines with IC_50_ = 11.05 and 29.09 μg/mL, respectively [50].	
Sarcostolide E **106**			Potent cytotoxic activity toward HeLa and WiDr cell lines with IC_50_ = 16.75 and 27.48μg/mL, respectively, and Daoy with IC_50_ = 5.5 μg/mL [50].	
Sarcostolide F **107**			Potent cytotoxic activity toward HeLa and WiDr cell lines with IC_50_ = 7.32 and 28.84 μg/mL, respectively [50].	
Sarcostolide G **108**			Potent cytotoxic activity toward HeLa and WiDr cell lines with IC_50_ = 18.45 and 20.06 μg/mL, respectively [50].	
(-)-7β-Hydroxy-8α-methoxy-deepoxy-sarcophytoxide **110**	*S. mililatensis*		Significant increase in the ALP activity collagen synthesis [51].	Baycanh Island, Condao District, Baria-Vungtau Province, Vietnam
(+)-7β,8β-Dihydroxy-deepoxy-sarcophytoxide **111**				
(-)-17-Hydroxysarcophytonin A **112**				
Sarcophytol V **113**				
Sarcophytoxide **114**	*S. mililatensis* *S. glaucum* *Sarcophyton* sp. *S. trocheliophorum*	Diterpene	Significant increase in the ALP activity collagen synthesis. Strong activity toward MCF-7 and HCT116 cells with IC_50_ = 9.9 ± 0.03 and 25.8 ± 0.03 µM, respectively. Activity toward Hep G2, Hep 3B, MDA-MB-231, A549, and Ca9-22 cell lines with IC_50_ = 16.2, 12.4, 13.2, 15.3, and 18.9 µg/mL, respectively [51,55,78].	Baycanh Island, Condao District, Baria-Vungtau Province, Vietnam. Red Sea, Jeddah, Saudi Arabia
7β-Acetoxy-8α-hydroxydeepoxy-sarcophine **116**	*S. glaucum*		Potent cytotoxicity toward HepG2, HCT-116, and HeLa cells with IC_50_ = 3.6, 2.3, and 6.7 μg/mL, respectively [65].	Hurghada, Red Sea, Egypt
7α,8β-Dihydroxydeepoxysarcophine **117**	*S. elegans* *S. auritum* *S. glaucum*		Cytotoxic activity toward A2780 cell line with IC_50_ > 10 μg/mL and against both breast and liver cancer cell lines with IC_50_ = 18.4 ± 0.16, 11 ± 0.22 µg/mL, respectively. Significantly decrease the viability of melanoma cells at 500 (72 hr) treatment., does not show toxic effect CV-1 cells and decrease de novo DNA synthesis and PARP activity [75,136].	Xidao Island, Hainan, China. Xidao Island, Hainan, China. Safaga Red Sea, Egypt
*Ent*-sarcophine **122**	*S. glaucum*		Potent suppression of the phase I enzyme cytochrome P450 1A activity with IC_50_ = 3.4 µM [66].	Yalong Bay, Hainan Province, China
Lobohedleolide **125**	*Sarcophyton* sp.		Most potent, inhibited the colony formation of Chinese hamster V79 at ED_50_ = 4.6 µM and decreased the TNFα-production at 3.0–10.0 µM [54].	Manado, North Sulawesi
(7Z)- Lobohedleolide **126**			Most potent, inhibited the colony formation of Chinese hamster V79 at ED_50_ = 4.6 µM and decreased the TNFα-production at 3.0–10.0 µM [54].	
7-Acetyl-8-*epi*- sinumaximol G **131**	*S. ehrenbergi*		Cytotoxic activity against MCF-7 with IC_50_ range 22.39 to 27.12 µg/mL [56].	Hurghada, Red Sea, Egypt
8-E*pi*- sinumaximol G **132**				
12-Acetyl-7, 12-*epi*- sinumaximol G **133**				
12-Hydroxysarcoph-10-ene **134**				
8-Hydroxy-*epi*-sarcophinone **135**				
Sinumaximol G **136**				
Sarcocrassocolide A **137**	*S. crassocaule*	Diterpene	Potent cytotoxic activity toward MCF-7, WiDr, HEp-2, and Daoy cancer with IC_50_ = 4.2, 3.2, 2.0, and 4.1 µg/mL, respectively. Decreased the levels of iNOS protein to 13.7 ± 5.2% at a concentration of 10 µM [58].	Dongsha Coast, Taiwan
Sarcocrassocolide B **138**			Potent cytotoxic activity toward MCF-7, WiDr, HEp-2, and Daoy cancer with IC_50_ = 4.2, 3.2, 1.2, and 1.8 µg/mL, respectively. Significantly decreased the levels of iNOS protein to 3.3 ± 5.0% at a concentration of 10 µM [58].	
Sarcocrassocolide C **139**			Potent cytotoxic activity toward MCF-7, WiDr, HEp-2, and Daoy cancer with IC_50_ = 6.2, 4.5, 2.6, and 4.0 µg/mL, respectively. Decrease significantly iNOS protein levels to 4.6 ± 1.3% at a concentration of 10 µM [58].	
Sarcocrassocolide D **140**			Potent cytotoxic activity toward MCF-7, WiDr, HEp-2, and Daoy cancer with IC_50_ = 8.8, 5.6, 3.2, and 5.4 µg/mL, respectively. Decrease significantly iNOS protein levels to 7.0 ± 3.1% at a concentration of 10 µM [58].	
Sarcocrassocolide F **143**			Potent toward MCF-7 cells with ED_50_ = 19.4 ± 2.4 μM. Decreased iNOS protein levels [59].	
Sarcocrassocolide G **144**			Potent toward Daoy, HEp-2 and WiDr cells with ED_50_ = 8.3 ± 1.4, 16.5 ± 1.7 and 18.9 ± 1.9 μM, respectively. Decreased iNOS protein levels [59].	
Sarcocrassocolide H **145**			Most potent toward MCF-7 ED_50_ = 9.4 ± 2.5 μM. Significantly suppressed both iNOS and COX-2 proteins expression [59].	
Sarcocrassocolide I **146**			Most potent toward Daoy, HEp-2, MCF-7, and WiDr cell lines with ED_50_ = 5.1 ± 1.2, 5.8 ± 0.5, 8.4 ± 1.5, and 6.4 ± 2.0 μM. Decreased iNOS protein levels [59].	
Sarcocrassocolide J **147**			Least potent toward Daoy, HEp-2, MCF-7, and WiDr cell lines with ED_50_ = >20 μM. Decreased iNOS protein levels [59].	
Sarcocrassocolide L **149**	*S. crassocaule*	Diterpene	Least potent toward Daoy, HEp-2, MCF-7, and WiDr cell lines with ED_50_ = >20 μM. Reduced iNOS protein levels [59].	Dongsha Coast, Taiwan
Sarcocrassocolide M **150**			Potent cytotoxicity toward Daoy, HEp-2, MCF-7, and WiDr with IC_50_ = 6.6 ± 0.8, 5.2 ± 0.6, and 5.0 ± 0.7 μM, respectively. Significantly decreased iNOS protein levels and COX-2 expression to 4.2 ± 1.6% and 62.8 ± 22.4%, respectively [60].	
Sarcocrassocolide N **151**			Potent cytotoxicity toward Daoy, HEp-2, MCF-7, and WiDr with IC_50_ = 10.4 ±1.1, 12.3 ± 1.6, and 12.4 ± 2.1 μM, respectively. Significantly decreased iNOS protein levels to 52.9 ± 12.8% [60].	
Sarcocrassocolide O **152**			Potent cytotoxicity toward Daoy, HEp-2, MCF-7, and WiDr with IC_50_ = 10.6 ± 0.5, 10.1 ± 2.3, and 6.4 ± 0.5 μM, respectively. Significantly decreased the levels of iNOS protein to 22.7 ± 2.8% [60].	
Sarcocrassocolide P **153**			Potent cytotoxic against DLD-1 and HL-6 (IC_50_ = 21.8 and 24.9 µM, respectively. Strongly reduced iNOS protein levels with 1.3% ± 0.3% [61].	
Sarcocrassocolide Q **154**			Potent cytotoxic toward only HL-6 (IC_50_ = 18.6 µM). Decreased iNOS protein levels and COX-2 expression with 2.4% ± 0.4% and 58.3% ± 20.5, respectively [61].	
Sarcocrassocolide R **155**			Potent cytotoxicity toward DLD-1, CCRF-CEM, and HL-60 cancer cells (IC_50_ = 10.0, 3.8, and 7.9 µM, respectively). Strongly reduced iNOS protein levels to 1.2% ± 0.3% [61].	
(+)-12-Carboxy-11Z-sarcophytoxide **157**	*S. ehrenbergi*		Antiviral activity toward HCMV with IC_50_ = 180.7 µM [63].	
(+)-12-Methoxycarbonyl-11Z-sarcophine **158**	*S. ehrenbergi*	Diterpene	Antiviral activity toward HCMV with IC_50_ = 5.8, 24.2, 24.8, 4.7, and 16.1 µM, respectively [63].	Dongsha Atoll off Taiwan
Ehrenberoxide A **159**				
Ehrenberoxide B **160**				
Ehrenberoxide C **161**				
Lobophynin C **162**				
Cembrene C **163**	*S. trocheliophorum*		Mild antidiabetic activity with IC_50_ = 26.6 µM. Antifungal activity toward *Aspergillus flavus* and *Candida albicans* (MIC = 0.68 µM) [39,77].	Yalong Bay, Hainan Province, China. Red Sea, Jeddah, Saudi Arabia
Sarcophytol B **164**	*Sarcophyton* sp.		Potent antibacterial activity toward *Bacillus cereus*, *Staphylococcus albus*, and *Vibrio parahaemolyticus* (MIC = 3.13, 1.56, and 0.50 μM, respectively) [73].	Xuwen Coral Reef Area, Guangdong Province, China
Sarcophytol H **166**	*S. infundibuliforme*		Strong antifouling activity toward the larval settlement of barnacle *Balanus Amphitrite* (EC_50_ = 8.13 µg/mL) [64].	Wenchang Coral Reef in the South China Sea
(–)-Marasol **167**	*S. infundibuliforme* *S. glaucum*		Antifouling activity on larval adherence of the barnacle *Balanus Amphitrite* at concentration of 10.0 µg/mL [73].	Xuwen Coral Reef, Guangdong Province, China
12(*S*)-Hydroperoxylsarcoph-10-ene **170**	*S. glaucum*		Potent suppression of the phase I enzyme cytochrome P450 1A activity with IC_50_ = 2.7 µM [66].	Yalong Bay, Hainan Province, China
8-*Epi*-sarcophinone **171**			Potent suppression of the phase I enzyme cytochrome P450 1A activity with IC_50_ = 3.7 µM [66].	
Methyl sarcotroate B **173**	*S. trocheliophorum*		Strong inhibitory activity toward PTP1B with IC_50_ = 6.97 μM [67].	
(1S,2E,4R,6E,8S,11R,12S)-8,11-Epoxy-4,12-epoxy-2,6-cembradiene **175**	*S. glaucum*		Cytotoxic activity at 500 µM concentration toward mouse melanoma B16F10 cells [68].	Red Sea
(1S,4R,13S)-Cembra-2E,7E,11E-trien-4,13-diol **177**				
Ehrenbergol B **179**	*S. ehrenbergi*		Strong antiviral activity with IC_50_ = 5 µg/mL [69].	San-Hsian-Tai, Taitong County, Taiwan
Sarcophyolide B **181**	*S. elegans*	Diterpene	Most potent cytotoxic activity toward A2780 with IC_50_ = 2.92 μM [22].	Xidao Island, Hainan, China
Sarcophyolide C **182**			Cytotoxic activity toward A2780 cell line with IC_50_ > 10 μg/mL [22].	
Sarcophyolide D **183**				
Sarcophyolide E **184**				
Sarcophytol L **185**				
13α-Hydroxysarcophytol L **186**				
Sarcophyolide A **187**				
Sarcophinone **188**				
7α-Hydroxy-Δ^8(19)^-deepoxysarcophine **189**				
4β-Hydroxy-Δ^2(3)^-sarcophine **190**				
1,15β-Epoxy-2-*epi*-16-deoxysarcophine **191**				
Sarcophytol Q **192**				
Lobocrasol **193**			Most potent cytotoxic activity toward A2780 cell line with IC_50_ = 3.37 μM [22].	
Acetyl ehrenberoxide B **194**	*S. ehrenbergi*		Antiviral activity toward HCMV with IC_50_ = 8 µg/mL [72].	San-Hsian-Tai, Taitong County, Taiwan
Ehrenbergol C **195**			Antiviral activity toward HCMV with IC_50_ = 20 µg/mL [72].	
Tortuosene A **201**	*S. tortuosum*		Potent inhibition 56.0 ± 3.1% against FMLP/CB-induced superoxide anion generation [74].	Lanyu Coast Island of Taiwan
Tortuosene B **202**			Mild inhibition of the elastase release 13.7 ± 3.5 [74].	
Sarcotrocheliol acetate **213**	*S. glaucum* *S. trocheliophorum*		Strong activity against HepG2 and MCF-7 cells with IC_50_ = 19.9 ± 0.02 and 2.4 ± 0.04 µM, respectively. Strong antibacterial activity with inhibition zones range (12 to 18 mm) and MICs between 1.53 to 4.34 µM, toward *Staphylococcus aureus, Acinetobacter* sp., and MRSA [77,78].	Red Sea, Jeddah, Saudi Arabia
Sarcotrocheliol **214**	*S. glaucum*	Diterpene	Strong antibacterial activity with inhibition zones range from 12 to 18 mm and MICs between 1.53 and 4.34 µM, toward Staphylococcus aureus, *Acinetobacter* sp., and MRSA. Strong activity toward MCF-7 cells with IC50 = 3.2 ± 0.02 µM, respectively [77,78].	Red Sea, Jeddah, Saudi Arabia
Sarcophinediol **215**			Strong activity against HepG2 and HCT116 with IC_50_ = 18.8 ± 0.07 and 19.4 ± 0.02 µM, respectively [78].	
2-[(E,E,E)-7′,8′-Epoxy-4′,8′,12′- trimethylcyclotetradeca-1′,3′,11′-trienyl]propan-2-ol **209**	*Sarcophyton* sp.		Mild inhibition more than 10% at a concentration of 20 μM toward the MCF-7 cell line [76].	Dongshan island, China
Crassumol C **211**				
Laevigatol A **212**				
Sarsolilide B **220**	*S. trocheliophorum*		Inhibited protein tyrosine phosphatase 1B IC_50_ = 6.8 ± 0.9 μM [80].	Yalong Bay, Hainan Province, China
Sarsolilide C **221**			Inhibited protein tyrosine phosphatase 1B IC_50_ = 27.1 ± 2.6 μM [81].	
Trocheliophol E **228**			Mild inhibition toward inflammation-related NF-kB by 11% [81].	Weizhou Island, Southwestern China
Trocheliophol F **229**			Mild inhibition toward inflammation-related NF-kB by 29% [81].	
Trocheliophol H **231**			Antibacterial activity toward *Xanthomonas vesicatoria*, *Agrobacterium tumefaciens*, *Pseudomonas lachrymans*, *Bacillus subtilis*, and *Staphylococcus aureus*, with MIC = 8 to 32 µg/mL [81].	
Trocheliophol I **232**				
Trocheliophol L **235**			Antibacterial activity toward *Xanthomonas vesicatoria*, *Agrobacterium tumefaciens*, *Pseudomonas lachrymans*, *Bacillus subtilis*, and *Staphylococcus aureus,* with MIC = 8 to 32 µg/mL [81].	
Trocheliophol M **236**			Mild inhibition toward inflammation-related NF-kB by 14% [81].	
Trocheliophol N **237**			Antibacterial activity toward *Xanthomonas vesicatoria*, *Agrobacterium tumefaciens*, *Pseudomonas lachrymans*, *Bacillus subtilis*, and *Staphylococcus aureus*, with MIC = 8 to 32 µg/mL [81].	
Trocheliophol O **238**	*S. trocheliophorum*	Diterpene	Antibacterial activity toward *Xanthomonas vesicatoria*, *Agrobacterium tumefaciens*, *Pseudomonas lachrymans, Bacillus subtilis,* and *Staphylococcus aureus,* with MIC = 8 to 32 µg/mL [81].	Weizhou Island, Southwestern China
Trocheliophol R **241**				
Trocheliophol S **242**			Most potent antibacterial activity against *Xanthomonas vesicatoria*, *Agrobacterium tumefaciens*, *Pseudomonas lachrymans, Bacillus subtilis,* and *Staphylococcus aureus* [81].	
4-Epi-sarcophytol L **243**			Antibacterial activity toward *Xanthomonas vesicatoria*, *Agrobacterium tumefaciens*, *Pseudomonas lachrymans*, *Bacillus subtilis*, and *Staphylococcus aureus,* with MIC = 8 to 32 µg/mL [81].	
Sarcophelegan B **245**	*S. elegans*		Significant cytotoxicity toward breast cancer MDA-MB-231 migration in a time dependent manner [18].	Xisha Islands, South China Sea
Ehrenbergol D **251**	*S. ehrenbergi*		Potent cytotoxic activity P-388 cell line with EC_50_ = 2.0 μM. Significant TNF-α inhibition IC_50_ = 24.2 μM [82,97].	San-Hsian-Tai Island (Taitong)
Ehrenbergol E **252**			Potent cytotoxic activity P-388 cell line with EC_50_ = 3.0 μM [82].	
Secodihydrosarsolenone **287**	*S. trocheliophorum*		Restrained activity toward PTP1B with IC_50_ = 13.7 µmol/L [92].	The South China Sea Coral Reef
Sarelengan C **297**	*S. elegans*		Significant inhibitory action on nitric oxide synthesis in RAW264.7 macrophages, with IC_50_ = 32.5 µM [94].	Yalong Bay, Hainan Province, China
Sarcoehrenbergilid D **307**	*S. ehrenbergi*		Strong cytotoxicity against A549 cells with IC_25_ = 23.3 μM [96].	Hurghada, Red Sea, Egypt
Sarcoehrenbergilid E **308**			Strong cytotoxicity activity against A549 and HepG2 cells with IC_25_ = 27.3 and 22.6 μM, respectively [96].	
Sarcoehrenbergilid F **309**			Strong cytotoxic activity against A549 cells with IC_25_ = 25.4 μM [96].	
Sarcoehrenolide A **310**			Significant TNF-α inhibition IC_50_ = 28.5 μM [97].	Weizhou Island, Guangxi Province, China
Sarcoehrenolide B **311**			Significant TNF-α inhibition IC_50_ = 8.5 μM [97].	
Sarcoehrenolide D **313**			Significant TNF-α inhibition IC_50_ = 27.3 μM [97].	
Sarinfacetamide A **315**	*S. infundibuliforme*	Diterpene	Increase effects of the ConA-induced T lymphocytes with 6.18% and 36.32% proliferation rates, respectively [98].	Ximao Island, Hainan Province, China
Nanolobatin B **317**				
(1S,2E,4R,6E,8S,11S,12S)-11,12-Epoxy-8-hydroperoxy-4-hydroxy-2,6-cembradiene **318**	*Sarcophyton* sp.		Potent antibacterial activity toward pathogens as *Alteromonas* sp., *Cytophaga*-*Flavobacterium,* and *Vibrio* sp. from seaweed, with antibiosis index = 0.5, 1.25, and 1.75, respectively [99].	Bohey Dulang, Semporna, Sabah
Glaucumolides A and B **329**–**330**	*S. glaucum*	Biscembrane	Potent cytotoxicity toward HL-60 and CCRF-CEM cancer cell lines with IC_50_ = 6.6 ± 1.2, 3.8 ± 0.9, 5.3 ± 1.4, and 7.4 ± 1.5μg/mL, respectively. Strong inhibition against superoxide anion generation with IC_50_ = 2.79 ± 0.66 μM and 2.79 ± 0.32 μM, respectively, and elastase release with IC_50_ = 3.97 ± 0.10 μM for both compounds and in vitro anti-inflammatory activity both significantly prevent the accumulation nitric oxide synthase protein [103].	From the wild and cultured in cultivation tank in the National Museum of Marine Biology and Aquarium, Taiwan
Bislatumlide A and B **340**–**341**	*S. latum*		Potent activity against A549 and WiDr tumor cell with IC_50_ = 7 µg/mL and murine lymphocytic leukemia with IC_50_ = of 5.8 µg/mL [105].	Ximao Island, Hainan Province, China
Methyl tetrahydrosarcoate **342**	*S. elegans*		Lethality bioassay exhibited IC_50_ = 1.5 μM [106].	Kitangambwe, Kenya
Dioxanyalolide **347**			Antimicrobial activity toward *Escherichia coli*. Lethality bioassay exhibited IC_50_ = 1.5 μM [106].	
Sarelengan B **363**			Significant inhibitory action on nitric oxide synthesis in RAW264.7 macrophages, with IC_50_ = 18.2 µM [94].	Yalong Bay, Hainan Province, China
(+)-alloaromadendrene **364**	*S. glaucum*	Sesquiterpene	Most potent with IC_50_ = 20.0 ± 0.068, 20.0 ± 0.054, and 09.3 ± 0.164 µM toward HepG2, MCF-7, and PC-3, respectively. Significant inhibition to +SA mammary epithelial cell growth [34].	North of Jeddah, Saudi Arabia, Red sea
Palustrol **370**	*S. trocheliophorum*		Potent activity toward Lymphoma and Erlish cell lines with LD_50_ range from 2.5 to 3.79 µM [77].	Red Sea, Jeddah, Saudi Arabia
6-Oxo-germacra-4(15),8,11-triene **373**	*S. glaucum*		Strong activity against HCT116 with IC_50_ = 25.8 ± 0.03 µM [78].	
23,24-Dimethylcholest-16(17)-E-ene-3β,5α,6β,20(S)-tetraol **374**	*S. trocheliophorum*	Sterol	Strong cytotoxicity toward human M14, HL60, and MCF7 cells (EC_50_ = 4.3, 2.8, and 4.9 µg/mL, respectively), with a dose-dependent manner [29].	Pulau Hantu Island, South Singapore
24-Methylcholestane-3β,5α,6β,25-tetraol-25-monoacetate **375**	*S. crassocaule* *S. glaucum* *S. trocheliophorum*		Potent activity toward the P-388, A549, and HT-29 cell lines with cell line with ED_50_ = 3.96, 6.6, and 0.6 µg/mL, respectively. Strong cytotoxicity against M14, HL60, and MCF7 cells with EC_50_ = 19.6, 13.2, and 34.5 µg/mL, respectively, with a dose-dependent manner [28].	Green Island, off Taiwan. Pulau Hantu Island, South Singapore
(24S)-24-Methylcholestane-3β,5α,6β-triol **377**	*S. crassocaule*		Potent activity toward the P-388 cell line with ED_50_ = 0.14 µg/mL, respectively [28].	Green Island, off Taiwan
Sardisterol **378**	*S. ehrenbergi*		Potent activity against A-549 cell line with IC_50_ = 27.3 μM [95].	Hurghada, Red Sea, Egypt
11α-Acetoxy-cholesta-24-en-3β,5α,6β-triol **384**	*Sarcophyton* sp.		Potent toward antibacterial activity toward *Escherichia coli* and and *Bacillus megaterium*, and antifungal activity toward *Microbotryum violaceum* and *Septoria tritici* fungi [115].	The coast of Weizhou Island, Guangxi Province of China
(22*E*,24*S*)-11α-Acetoxy-ergostane-22,25-dien-3β,5α,6β-triol **385**				
11α-Acetoxy-gorgostane-3β,5α,6β,12α-tetraol **389**				
12α-Acetoxy-gorgostane-3β,5α,6β,11α-tetraol **390**				
Sarcoaldosterol A **391**				
Sarcoaldesterol B **396**	*S. glaucum*		Cytotoxicity toward HepG2, MDA-MB-231, and A-549 cell lines with IC_50_ = 9.7, 14.0, and 15.8 µg/mL, respectively [70].	Jihui Fishing Port Coast, Taitung county, Taiwan
(24S)-Ergostan-3β,5α,6β,25-tetraol 25-monoacetate **398**	*Sarcophyton* sp.		Potent cytotoxic toward K562 with IC_50_ = 12.30 µg/mL [116].	Xuwen Coral Reef, South China Sea
(24S)-24-methylcholestan-3β,6β,25-triol-25-O-acetate **399**			Potent activity toward *Staphylococcus albus* with MIC = 20 µg/mL [116].	
(24S)-24-Methylcholestan-1β,3β,5α,6β,25-pentaol-25-monoacetate **401**			Potent cytotoxicity toward K562 with IC_50_ = 4.95 µg/mL. Potent activity toward *Staphylococcus albus* with MIC = 20 µg/mL [116].	
(24S)-Methylcholestan-3β,5α,6β,12β,25-pentaol-25-O-acetate **402**	*Sarcophyton* sp.	Sterol	Potent cytotoxic toward K562 with IC_50_ = 4.10 µg/mL [116].	Xuwen Coral Reef, South China Sea
(24S)-Ergostan-3β,5α,6β,18,25-pentaol 18,25-diacetate **403**			Potent cytotoxic toward K562 with IC_50_ = 5.25 µg/mL [116].	
Zahramycin B **405**	*S. trocheliophorum*		Potent antimicrobial (15 mm) and (12 mm) activity toward *Staphylococcus aureus* and *Bacillus subtilis*, respectively, and potent activity toward *Pythium ultimum* pathogenic fungus (12 mm) [117].	Hurghada, Red Sea, Egypt
(23*R*,24*R*,17*Z*)-11α-Acetoxy-16β-methoxy-23,24-dimethylcholest-17(20)-en-3β,5α,6β-triol **406**	*Sarcophyton* sp.		Strong cytotoxic activity against K562, HL-60, and HeLa cell lines with IC_50_ range of 6.4 to 24.7 μM [118].	South Sea, Weizhou Islands
11α-Acetoxycholest-24-en-1α,3β,5α,6β-tetraol **408**			Potent activity toward K562 and HL-60 with IC_50_ range of 9.1 to 17.2 μM [118].	
(24*R*)-Methylcholest-7-en-3β,5α,6β-triol **409**			Potent anti-H_1_N_1_ virus activity with IC_50_ = 19.6 μg/mL [118].	
11α-Acetoxy-cholest-24-en-3β,5α,6β-triol **410**			Potent activity toward K562 and HL-60 with IC_50_ range of 9.1 to 17.2 μM [118].	
(22*E*,24*S*)-11α-Acetoxy-ergost-22, 25-dien-3β,5α,6β-triol **411**			Strong cytotoxicity against, K562, HL-60, HeLa cell lines with IC_50_ range of 6.4 to 24.7 μM [118].	
(24*S*)-11α-Acetoxy-ergost-3β,5α,6β-triol **412**			Potent activity toward K562 and HL-60 with IC_50_ range of 9.1 to 17.2 μM [118].	
(24*R*)-11α-Acetoxy-gorgost-3β,5α,6β-triol **413**			Strong cytotoxicity toward, K562, HL-60, HeLa cell lines with IC_50_ range of 6.4 to 24.7 μM [118].	
(24*S*)-Ergost-3β,5α,6β,11α-tetraol **414**			Potent anti-H_1_N_1_ virus activity with IC_50_ = 36.7 μg/mL [118].	
Sarcomilasterol **419**	*S. glaucum*		Cytotoxicity toward MDA-MB-231, MOLT-4, SUP-T, and U-937 cell lines with IC_50_ = 13.8, 6.7, 10.5, and 17.7 µg/mL, respectively [70].	Jihui Fishing Port Coast, Taitung County, Taiwan
Butenolides **430**–**433**	*S. trocheliophorum*	Miscellaneous	Active against gram positive bacteria only [122].	Gulf of Aqaba, Tel Aviv
Sarcophytonamine **440**	*S. crassocaule*		Protection against UV radiation for organism [124].	Lingshui Bay, Hainan Province, China
Ceramide **469**	*S. ehrenbergi* *S. auritum*	Miscellaneous	Decreased iNOs to 46.9 ± 9.7% and COX-2 level to 77.2 ± 9.9%. Anticonvulsant activity, successfully opposed the lethality of pentylenetetrazole in mice. Significant anxiolytic activity [129,135].	Dongsha Islands, Taiwan Red Sea
Methyl tortuoate A **476**	*S. tortuosum*		Strong cytotoxic activity toward CNE-2 and P-388 cell lines with IC_50_ = 22.7, 3.5, 24.7, and 5.0 *µ*g/mL, respectively [131].	Sanya Bay, Hainan Island, China
Methyl tortuoate B **477**			Strong cytotoxic activity toward CNE-2 and P-388 cell lines with IC_50_ = 24.7 and 5.0 *µ*g/mL, respectively [131].	
Methyl sartortuoate **478**	*S. pauciplicatum*		Good cytotoxic activity toward HepG2, HL-60, KB, LNCaP, LU-1, MCF7, SK-Mel2, and SW480 cancer cells with IC_50_ ranged from 7.93 ± 2.08 to 19.34 ± 0.72 µM [108].	Hai Phong, Vietnam

## Supplementary Files

Supplementary File 1
