# Supplementary material for: Chemical Diversity in Species Belonging to Soft Coral Genus Sacrophyton and Its Impact on Biological Activity: A Review
Source: Mar Drugs. 2020 Jan 6;18(1):41. doi: 10.3390/md18010041 (PMC7024209; doi:10.3390/md18010041)
Supplement: Supplementary File 1 [file marinedrugs-18-00041-s001.pdf]

Supplementary

Chemical Diversity in Species Belonging to Soft Coral Genus *Sacrophyton* and Its Impact on Biological Activity: A Review

Yasmin A. Elkhawas, Ahmed M. Elissawy, Mohamed S. Elnaggar, Nada M. Mostafa, Eman Al-Sayed, Mokhtar M. Bishr, Abdel Nasser B. Singab and Osama M. Salama

Table S1: Listing compounds with PubChem CID number listed in Table 1

Compound name	PubChem
13-Acetoxysarcocrassolide	https://pubchem.ncbi.nlm.nih.gov/compound/21775908
Sarcophin	https://pubchem.ncbi.nlm.nih.gov/compound/6436805
Sarcophytol A	https://pubchem.ncbi.nlm.nih.gov/compound/5284454
Sarcoglaucol-16-one	https://pubchem.ncbi.nlm.nih.gov/compound/10361171
Decaryiol	https://pubchem.ncbi.nlm.nih.gov/compound/101658812
Sarcophytolide	https://pubchem.ncbi.nlm.nih.gov/compound/11381519
Yalongene A	https://pubchem.ncbi.nlm.nih.gov/compound/56964359
Sarcrassin D	https://pubchem.ncbi.nlm.nih.gov/compound/44559143
Sarcrassin E	https://pubchem.ncbi.nlm.nih.gov/compound/16091635
Emblide	https://pubchem.ncbi.nlm.nih.gov/compound/44559144
Crassocolide A	https://pubchem.ncbi.nlm.nih.gov/compound/11995173
Crassocolide B	https://pubchem.ncbi.nlm.nih.gov/compound/11995280
Crassocolide D	https://pubchem.ncbi.nlm.nih.gov/compound/11995281
Crassocolide E	https://pubchem.ncbi.nlm.nih.gov/compound/11995175
Crassocolide F	https://pubchem.ncbi.nlm.nih.gov/compound/11995282
Crassocolide N	https://pubchem.ncbi.nlm.nih.gov/compound/54764414
Crassocolide O	https://pubchem.ncbi.nlm.nih.gov/compound/54764415
Crassocolide P	https://pubchem.ncbi.nlm.nih.gov/compound/46930683
Sarcostolide A	https://pubchem.ncbi.nlm.nih.gov/compound/24970448
Sarcostolide B	https://pubchem.ncbi.nlm.nih.gov/compound/24970449
Sarcostolide C	https://pubchem.ncbi.nlm.nih.gov/compound/24970507

Sarcostolide D	https://pubchem.ncbi.nlm.nih.gov/compound/24970450
Sarcostolide E	https://pubchem.ncbi.nlm.nih.gov/compound/102394818
Sarcostolide F	https://pubchem.ncbi.nlm.nih.gov/compound/24970506
Sarcostolide G	https://pubchem.ncbi.nlm.nih.gov/compound/24970509
(-)-17-Hydroxysarcophytonin A	https://pubchem.ncbi.nlm.nih.gov/compound/101863296
Sarcophytol V	https://pubchem.ncbi.nlm.nih.gov/compound/101863297
Sarcophytoxide	https://pubchem.ncbi.nlm.nih.gov/compound/6440443
Lobohedleolide	https://pubchem.ncbi.nlm.nih.gov/compound/6475569
(7Z)- Lobohedleolide	https://pubchem.ncbi.nlm.nih.gov/compound/6475568
Sarcocrassocolide A	https://pubchem.ncbi.nlm.nih.gov/compound/70691856
Sarcocrassocolide B	https://pubchem.ncbi.nlm.nih.gov/compound/70696020
Sarcocrassocolide C	https://pubchem.ncbi.nlm.nih.gov/compound/46233394
Sarcocrassocolide D	https://pubchem.ncbi.nlm.nih.gov/compound/46233452
Lobophynin C	https://pubchem.ncbi.nlm.nih.gov/compound/10760017
Cembrene C	https://pubchem.ncbi.nlm.nih.gov/compound/14336535
Sarcophytol B	https://pubchem.ncbi.nlm.nih.gov/compound/6438700
(–)-Marasol	https://pubchem.ncbi.nlm.nih.gov/compound/100921067
Methyl sarcotroate B	https://pubchem.ncbi.nlm.nih.gov/compound/71520813
Sarcophinone	https://pubchem.ncbi.nlm.nih.gov/compound/15523779
Lobocrasol	https://pubchem.ncbi.nlm.nih.gov/compound/101485781
Sarcotrocheliol acetate	https://pubchem.ncbi.nlm.nih.gov/compound/132544619
Laevigatol A	https://pubchem.ncbi.nlm.nih.gov/compound/52920790
Sarsolilide B	https://pubchem.ncbi.nlm.nih.gov/compound/75593390
Sarsolilide C	https://pubchem.ncbi.nlm.nih.gov/compound/102141812
Trocheliophol H	https://pubchem.ncbi.nlm.nih.gov/compound/122206504
Trocheliophol E	https://pubchem.ncbi.nlm.nih.gov/compound/122206502
Trocheliophol F	https://pubchem.ncbi.nlm.nih.gov/compound/122206503
Trocheliophol I	https://pubchem.ncbi.nlm.nih.gov/compound/122206505
Trocheliophol L	https://pubchem.ncbi.nlm.nih.gov/compound/122206506
Trocheliophol M	https://pubchem.ncbi.nlm.nih.gov/compound/122206507
Trocheliophol N	https://pubchem.ncbi.nlm.nih.gov/compound/122206508
Trocheliophol O	https://pubchem.ncbi.nlm.nih.gov/compound/122206511
Trocheliophol R	https://pubchem.ncbi.nlm.nih.gov/compound/122206509
Trocheliophol S	https://pubchem.ncbi.nlm.nih.gov/compound/122206510

Ehrenbergol D	https://pubchem.ncbi.nlm.nih.gov/compound/122379341
Ehrenbergol E	https://pubchem.ncbi.nlm.nih.gov/compound/122379341
Nanolobatin B	https://pubchem.ncbi.nlm.nih.gov/compound/10064493
Bislatumlide A	https://pubchem.ncbi.nlm.nih.gov/compound/102090379
Bislatumlide B	https://pubchem.ncbi.nlm.nih.gov/compound/102090380
Methyl tetrahydrosarcoate	https://pubchem.ncbi.nlm.nih.gov/compound/44445649
Dioxanyalolide	https://pubchem.ncbi.nlm.nih.gov/compound/101838240
(+)-alloaromadendrene	https://pubchem.ncbi.nlm.nih.gov/compound/21775889
Palustrol	https://pubchem.ncbi.nlm.nih.gov/compound/110745
Tetradecyl octadecenoate	https://pubchem.ncbi.nlm.nih.gov/compound/129730668
Isoneocembrene A	https://pubchem.ncbi.nlm.nih.gov/compound/6521442
(1S,2E,4R,6E,8S,11S,12S)-11,12-Epoxy-2,6-cembrane-4,8-diol	https://pubchem.ncbi.nlm.nih.gov/compound/10064750
(1S, 2E, 4R, 6E, 8R,11S,12S)-11,12-Epoxy-2,6-cembrane-4,8-diol	https://pubchem.ncbi.nlm.nih.gov/compound/10471391
Sarcophytonolide B	https://pubchem.ncbi.nlm.nih.gov/compound/101720914
Sarcophytonolide A	https://pubchem.ncbi.nlm.nih.gov/compound/101720913
Sarcophytonolide C	https://pubchem.ncbi.nlm.nih.gov/compound/71584108
Sarcophytonolide D	https://pubchem.ncbi.nlm.nih.gov/compound/101720915
Sarcophytonolide E	https://pubchem.ncbi.nlm.nih.gov/compound/11652681
Sarcophytonolide F	https://pubchem.ncbi.nlm.nih.gov/compound/11652644
Sarcophytonolide G	https://pubchem.ncbi.nlm.nih.gov/compound/11551473
Sarcophytonolide H	https://pubchem.ncbi.nlm.nih.gov/compound/44583835
Sarcophytonolide I	https://pubchem.ncbi.nlm.nih.gov/compound/23624835
Sarcophytonolide J	https://pubchem.ncbi.nlm.nih.gov/compound/23624833
Sarcophytonolide K	https://pubchem.ncbi.nlm.nih.gov/compound/23624836
Sarcophytonolide L	https://pubchem.ncbi.nlm.nih.gov/compound/23624834
Sarcophytonolide N	https://pubchem.ncbi.nlm.nih.gov/compound/71770297
Sarcophytonolide P	https://pubchem.ncbi.nlm.nih.gov/compound/71770417
Sarcophytonolide Q	https://pubchem.ncbi.nlm.nih.gov/compound/71770418
Ketoemblide	https://pubchem.ncbi.nlm.nih.gov/compound/73350688
Sartrolide A	https://pubchem.ncbi.nlm.nih.gov/compound/119079608
Sartrolide B	https://pubchem.ncbi.nlm.nih.gov/compound/119079609
Sartrolide C	https://pubchem.ncbi.nlm.nih.gov/compound/72198139
Sartrolide D	https://pubchem.ncbi.nlm.nih.gov/compound/72198325
Sartrolide E	https://pubchem.ncbi.nlm.nih.gov/compound/72198326

Sartrolide F	https://pubchem.ncbi.nlm.nih.gov/compound/119079610
Sartrolide G	https://pubchem.ncbi.nlm.nih.gov/compound/119079611
Bissartrolide dimer	https://pubchem.ncbi.nlm.nih.gov/compound/102107370
Yalongene B	https://pubchem.ncbi.nlm.nih.gov/compound/101568826
Sarcrassin C	https://pubchem.ncbi.nlm.nih.gov/compound/16091633
Crassocolide C	https://pubchem.ncbi.nlm.nih.gov/compound/11995174
Lobophytolide	https://pubchem.ncbi.nlm.nih.gov/compound/23425511
Flexusine A	https://pubchem.ncbi.nlm.nih.gov/compound/102393972
Flexusine B	https://pubchem.ncbi.nlm.nih.gov/compound/102393973
Epimukulol	https://pubchem.ncbi.nlm.nih.gov/compound/13255923
Isosarcophin	https://pubchem.ncbi.nlm.nih.gov/compound/6442773
sarcophytonin A	https://pubchem.ncbi.nlm.nih.gov/compound/23425614
Sarcophytonin F	https://pubchem.ncbi.nlm.nih.gov/compound/102195304
Sarcophytonin G	https://pubchem.ncbi.nlm.nih.gov/compound/71467237
Sinularolide	https://pubchem.ncbi.nlm.nih.gov/compound/44583942
(+)-7,8-epoxy-7,8-dihydrocembrene C	https://pubchem.ncbi.nlm.nih.gov/compound/101089772
Methyl sarcotroate A	https://pubchem.ncbi.nlm.nih.gov/compound/71520812
Sarcophytonolide M	https://pubchem.ncbi.nlm.nih.gov/compound/102448084
2-epi-Sarcophin	https://pubchem.ncbi.nlm.nih.gov/compound/14586904
Dihydrosarsolenone	https://pubchem.ncbi.nlm.nih.gov/compound/102141809
Methyl dihydrosarsolenone	https://pubchem.ncbi.nlm.nih.gov/compound/102141811
Sarsolilide A	https://pubchem.ncbi.nlm.nih.gov/compound/23427413
3,4-epoxyehrenberoxide A	https://pubchem.ncbi.nlm.nih.gov/compound/122379340
Sinugibberol	https://pubchem.ncbi.nlm.nih.gov/compound/15275756
Sarcophytol M	https://pubchem.ncbi.nlm.nih.gov/compound/86577759
Bisglaucumlide A	https://pubchem.ncbi.nlm.nih.gov/compound/16083073
Bisglaucumlide B	https://pubchem.ncbi.nlm.nih.gov/compound/16083074
Bisglaucumlide C-D	https://pubchem.ncbi.nlm.nih.gov/compound/101407764
Ximaolide A	https://pubchem.ncbi.nlm.nih.gov/compound/16756316
Ximaolide B	https://pubchem.ncbi.nlm.nih.gov/compound/16756435
Ximaolide C	https://pubchem.ncbi.nlm.nih.gov/compound/16756318
Ximaolide E	https://pubchem.ncbi.nlm.nih.gov/compound/102471327
Ximaolide F	https://pubchem.ncbi.nlm.nih.gov/compound/102026833
Ximaolide G	https://pubchem.ncbi.nlm.nih.gov/compound/102026834

Methyl tortuosoate A	https://pubchem.ncbi.nlm.nih.gov/compound/16756315
Isosarcophytonolide D	https://pubchem.ncbi.nlm.nih.gov/compound/102090378
Nyalolide	https://pubchem.ncbi.nlm.nih.gov/substance/103638018
Desacetylnyalolide	https://pubchem.ncbi.nlm.nih.gov/compound/101838241
Diepoxynyalolide	https://pubchem.ncbi.nlm.nih.gov/compound/24762830
Lobophytone H	https://pubchem.ncbi.nlm.nih.gov/compound/102054998
Dioxosarcoguaiacol	https://pubchem.ncbi.nlm.nih.gov/compound/53344582
Arachidonic acid	https://pubchem.ncbi.nlm.nih.gov/compound/444899
Eicosapentaenoic acid	https://pubchem.ncbi.nlm.nih.gov/compound/446284
Prostaglandin PGB2	https://pubchem.ncbi.nlm.nih.gov/compound/5280881
Sarcophytonone	https://pubchem.ncbi.nlm.nih.gov/compound/44514015
Sarcoglycoside A	https://pubchem.ncbi.nlm.nih.gov/compound/44254754
Sarcoglycoside B	https://pubchem.ncbi.nlm.nih.gov/compound/44254756
Sarcoglycoside C	https://pubchem.ncbi.nlm.nih.gov/compound/44254755
Chimyl alcohol	https://pubchem.ncbi.nlm.nih.gov/compound/72733
Hexadecanol	https://pubchem.ncbi.nlm.nih.gov/compound/2682
Sarcoehrenoside A	https://pubchem.ncbi.nlm.nih.gov/compound/45271338
Peridinin	https://pubchem.ncbi.nlm.nih.gov/compound/5289155
Peridininol	https://pubchem.ncbi.nlm.nih.gov/compound/101764618
Peridininol -5,8-furanoxide	https://pubchem.ncbi.nlm.nih.gov/compound/101776196
Methyl isosartortuoate	https://pubchem.ncbi.nlm.nih.gov/compound/101056950
Methyl tortuoate C	https://pubchem.ncbi.nlm.nih.gov/compound/11490894
Methyl tortuoate A	https://pubchem.ncbi.nlm.nih.gov/compound/11490894
Methyl tortuoate B	https://pubchem.ncbi.nlm.nih.gov/compound/11331392
Methyl sartortuoate	https://pubchem.ncbi.nlm.nih.gov/compound/101366102
